# Tunable Proton Conductivity and Color in a Nonporous Coordination Polymer via Lattice Accommodation to Small Molecules

**DOI:** 10.1002/advs.202102619

**Published:** 2021-10-18

**Authors:** Aysegul Develioglu, Esther Resines‐Urien, Roberta Poloni, Lucía Martín‐Pérez, Jose Sanchez Costa, Enrique Burzurí

**Affiliations:** ^1^ IMDEA Nanociencia Campus de Cantoblanco Calle Faraday 9 Madrid 28049 Spain; ^2^ CNRS SIMAP Université Grenoble Alpes Grenoble 38000 France

**Keywords:** coordination polymers, nonporous, proton transport, vapochromic materials

## Abstract

Nonporous coordination polymers (npCPs) able to accommodate molecules through internal lattice reorganization are uncommon materials with applications in sensing and selective gas adsorption. Proton conduction, extensively studied in the analogue metal‐organic frameworks under high‐humidity conditions, is however largely unexplored in spite of the opportunities provided by the particular sensitivity of npCPs to lattice perturbations. Here, AC admittance spectroscopy is used to unveil the mechanism behind charge transport in the nonporous **1**·2CH_3_CN. The conductance in the crystals is found to be of protonic origin. A vehicle mechanism is triggered by the dynamics of the weakly coupled acetonitrile molecules in the lattice that can be maintained by a combination of thermal cycles, even at low humidity levels. An analogue **1**·pyrrole npCP is formed by in situ exchange of these weakly bound acetonitrile molecules by pyrrole. The color and conduction properties are determined by the molecules weakly bonded in the lattice. This is the first example of acetonitrile‐mediated proton transport in an npCP showing distinct optical response to different molecules. These findings open the door to the design of switchable protonic conductors and capacitive sensors working at low humidity levels and with selectivity to different molecules.

## Graphical abstract

Proton conductivity mediated by acetonitrile molecules is unveiled in a nonporous coordination polymer. A vehicle mechanism is triggered by the dynamics of the weakly coupled acetonitrile molecules in the lattice that can be maintained by a combination of thermal cycles, even at low humidity levels. The color and conductivity are tuned by in situ replacement of acetonitrile by other small molecules like pyrrole.

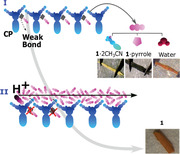

## 1 Introduction

Nonporous coordination polymers (npCPs) that can act as porous are remarkable materials able to release or incorporate small molecules in their structure via internal lattice reorganization.^[^ ^1^, ^2^, ^3^ ^]^ These molecules are typically weakly coupled to the lattice and can migrate across the crystal, providing npCPs with complex molecular dynamics and an associated wide variety of applications from magnetic switching,^[^ ^1^, ^3^, ^4^ ^]^ gas/vapor adsorption^[^ ^5^, ^6^, ^7^, ^8^ ^]^ and sensing,^[^ ^4^, ^9^, ^10^, ^11^ ^]^ energy storage,^[^ ^12^ ^]^ and catalysis,^[^ ^13^ ^]^ even though this is a relatively recent research area. Owing to the weak interactions in the crystal packing, these materials are usually softer than porous coordination polymers (pCP) and metal‐organic frameworks (MOF). This “softness” can allow npCPs to exhibit a greater affinity and sensitivity to adsorbate molecules^[^ ^1^, ^2^, ^3^ ^]^ compared to many MOFs for which ligand functionalization is often required.^[^ ^14^, ^15^, ^16^, ^17^ ^]^

Electron transport in coordination polymers in general has proven so far to be elusive. Only a few reports point in that direction for some specific MOFs.^[^ ^18^, ^19^, ^20^, ^21^ ^]^ An interesting alternative source for charge transport would be protons or more complex cations. Proton conduction in solids plays a fundamental role in fuel cells^[^ ^22^ ^]^ and biological systems.^[^ ^23^ ^]^ In contrast to typical amorphous acidic polymers, npCPs, MOFs, and pCPs can combine ion conductivity with a crystal‐like structure which in turn provides a handle to tune transport properties by proper chemical design. Extensive work, mainly in MOFs, has been performed in recent years to attain a clear picture describing proton transport in these materials as well as the role of ambient molecules like water.^[^ ^24^, ^25^ ^]^ Proton conductivity in MOFs typically stems from donor guest molecules in the pores^[^ ^26^, ^27^, ^28^, ^29^, ^30^, ^31^, ^32^ ^]^ or adhered water molecules,^[^ ^33^, ^34^, ^35^, ^36^, ^37^ ^]^ and can be tuned with acidic groups within the lattice structure^[^ ^33^, ^38^, ^39^, ^40^ ^]^ or by introducing networks of van der Waals interactions.^[^ ^41^ ^]^ Most of these examples work under large relative humidity (>90% RH) levels.

Unlike MOFs, the potential of npCPs to host proton transport is largely unexplored. Only a few examples, based on water‐assisted transport show their potential to compete with or even outperform some MOFs.^[^ ^42^, ^43^, ^44^, ^45^, ^46^ ^]^

**Figure 1:**
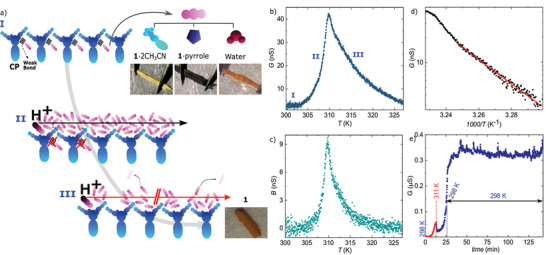
a) Schematics of the acetonitrile‐mediated vehicle proton transport: I) Acetonitrile is weakly bonded to the lattice. II) Acetonitrile is released enabling a transient path for proton vehicle transport. III) The evaporation of acetonitrile quenches the proton transport path. b) Conductance *G* and c) susceptance *B* measured as a function of the temperature *T* with a lock‐in technique in sample 1. A peak appears at *T*_*c*_(aceto) = 310 K. d) Arrhenius plot of *G* below *T*_*c*_(aceto). The activation energy is *E*_*a*_ = 1.28 eV, consistent with vehicle transport of protons. e) *G* measured in sample 2 as a function of *T* until 312 K, and back to room temperature. Thereafter the crystal is left to relax with time for 2 hours.

Recently, we reported a novel nonporous coordination polymer (_∞_[Fe(H_2_O)_2_(CH_3_CN)_2_(pyrazine)](BF_4_)_2_. (CH_3_CN)_2_ complex, hereafter **1**·2CH_3_CN).^[^ ^47^ ^]^ The Fe core is hexacoordinated with two water, two acetonitrile, and two pyrazine molecules forming closely packed 1D coordination chains. Besides, **1**·2CH_3_CN contains two uncoordinated acetonitrile molecules coupled to the polymer chains through hydrogen bonds formed with the coordinated water molecules. No weakly bonded water molecules are found in the lattice structure (see Section 1 in the Supporting Information for the structure). The **1**·2CH_3_CN crystals were able to reversibly modify their electro‐optical properties by desorption of the uncoordinated acetonitrile molecules from the crystal lattice^[^ ^47^ ^]^ (see schematics in **Figure** **1**a). A sharp peak in the DC electrical current was reported to coincide in temperature with a crystal phase transition and subsequent breaking of van der Waals bonds of interstitial acetonitrile molecules.^[^ ^47^ ^]^ The conductance peak was accompanied by a color change of the crystal from light yellow to orange. The mechanism able to translate the phase transition to a sharp change in the current was however not understood. The selectivity of the lattice to accommodate acetonitrile or other small volatile molecules was not explored.

Here, we use AC admittance spectroscopy to unveil the mechanism behind charge transport in the nonporous **1**·CH_3_CN. We show that the conductance in the crystals is of protonic origin. A vehicle transport mechanism is activated by the dynamics of weakly coupled acetonitrile molecules in the lattice that leads to a non‐trivial modification of the capacitance and resistance of the material. A simple combination of thermal cycles allows to trigger and maintain the conductance over time. We show that this acetonitrile‐based mechanism preserves proton transport even at low humidity levels (RH<40%), in contrast to most CPs. We have also exchanged in situ the uncoordinated acetonitrile molecules in the lattice by pyrrole, forming the new **1**·pyrrole. We show that the optical response (color) and the proton transport properties (critical temperature, conductance, etc) are determined by the nature of the molecules weakly bonded to the lattice, thanks to the particular sensitivity of npCPs to perturbations to their lattices, not easily achieved by more rigid MOFs. This is the first example of acetonitrile‐mediated proton transport in an npCP showing distinct optical response to different molecules.

## 2 Results and Discussion

Figure 1b,c shows the temperature *T* dependence of the real and imaginary components of the complex admittance Y* measured on a single crystal (sample 1) with a lock‐in technique. These complex components correspond respectively to the conductance *G* and the susceptance *B* of the material such that Y* = *G* + *iB*. The heating rate is set to +1 K min^‐1^ and the AC excitation is fixed to a frequency *ω*/2*π* = 10 kHz and an AC voltage *V*_*ac*_ = 100 mV. An offset DC bias voltage is set to *V* = 1 V. A sharp peak centered at *T*_*c*_(aceto) = 310 K appears in *G* by increasing the temperature, as previously observed in DC conductance measurements after the release of acetonitrile molecules, see schematics in Figure 1a.^[^ ^47^ ^]^ The conductance at the peak, normalized to the dimensions of the crystals is ≈10*μ*S cm^‐1^. These values are similar to those typically found in the literature^[^ ^42^, ^46^ ^]^ The initial increment in *G* can be fitted to an Arrhenius law for thermally activated transport (see Figure 1d and additional examples in Section S2, Supporting Information). The activation energy barrier obtained from the fit is *E*_*a*_= 1.28 eV. Thereafter, the thermally activated conductance quenches above *T*_*c*_(aceto) and *G* drops toward the initial background following a softer exponential decay with temperature. Note that no response is observed in *G* during cooling back to room temperature or in subsequent thermal cycles (see Figure S6, Supporting information). Optical and conductance reversibility are regained only once the crystal is exposed again to acetonitrile, as previously reported for DC measurements.^[^ ^47^ ^]^ The conductance is therefore intrinsically connected to the transient presence of free acetonitrile.

To disentangle the roles of temperature and time decay in the post‐transition relaxation, an additional control measurement (sample 2) is performed where the temperature is swept from room *T* to *T*_*c*_(aceto) (red points in Figure 1e) and ramped back to room *T* (blue points). Thereafter, *G* is measured as a function of time at room *T* (see Figure 1e). Initially, *G* slightly decreases at *T*_*c*_, most likely due to a thermal lag. Thereafter, no significant decay of *G* is observed in timescales (2 h) much larger than those employed in the temperature‐dependent measurement (12 min) in Figure 1b. The same behavior is observed by stopping the temperature ramp at *T*_*c*_ (see Figure S7, Supporting Information). The dynamics are therefore predominantly governed by the increment in temperature and not the time decay. Accordingly, a small thermal cycle allows to activate and maintain conductance in the crystal at room temperature.

The singular behavior of the admittance observed in these crystals can be explained in terms of proton transport, triggered by the particular dynamics of the acetonitrile molecules. The high *E*_*a*_ value discards a Grotthuss mechanism and is consistent with a vehicle transport mechanism where protons diffuse assisted by vehicle molecules in a liquid‐like environment. See schematics in Figure 1a. This mechanism is typically described for solvent‐assisted, typically water, proton transport in MOFs.^[^ ^33^ ^]^ The release of acetonitrile molecules from the crystal lattice seems to enable a transient channel for vehicle transport of protons (scheme II in Figure 1a). A similar mechanism has been described for other nonporous coordination polymers where the sudden rotational motion of small molecules in the lattice induces a non‐linear increase of the proton conductivity.^[^ ^46^ ^]^ The proton source in **1**·2CH_3_CN is possible moisture or coordinated water in the lattice. On the other hand, it has been recently shown that acetonitrile plays a fundamental role in the proton dynamics in zeolites. It is shown that a protonated solvent cluster may form where acetonitrile dimers [(CH_3_CN)_2_H]^+^ form under specific conditions of high acetonitrile pressure, temperature or in small pores.^[^ ^48^ ^]^ The subsequent temperature‐induced quenching of the conductance can be ascribed to the progressive evaporation of the acetonitrile molecules, that is, the vehicle molecules enabling proton transport, from the surface (see schematics III in Figure 1a). The vapor pressure, and thus the transfer rate of free molecules from surface to gas phase, grows exponentially with increasing temperature, as described by the Antoine semi‐empirical equation. A substantial evaporation of acetonitrile concomitant with the crystal phase transition has been demonstrated on videos recorded on large pellets of the compound.^[^ ^47^ ^]^ This evaporation can be reversed/frozen to maintain a constant vehicle path and therefore conductance, as seen in Figure 1e. We can safely discard the conduction of BF${}_{4}^{-}$ anions since that would lead to the disintegration of the crystals and the process would be irreversible, whereas we observe a clear reversibility in our crystals

Interestingly, the out‐of‐phase imaginary component *B* develops as well a clear peak at the same temperature (see Figure 1c). The shape of the peak evolves to an oscillatory perturbation around a constant background for higher AC frequencies (510 kHz), as shown in Figure S4, Supporting Information. This frequency and temperature perturbation in *B*, together with the progressive drop in *G*, cannot be understood in terms of a simple thermally activated transport mechanism through the crystal. The first, in particular, points to a non‐trivial evolution of the capacitive properties of the crystal at temperatures near the phase transition, due to the reorganization and loss of acetonitrile. A similar quantitative behavior is observed in 16 samples (80 % of the total) (see Section S3, Supporting Information, for detailed statistics).

**Figure 2:**
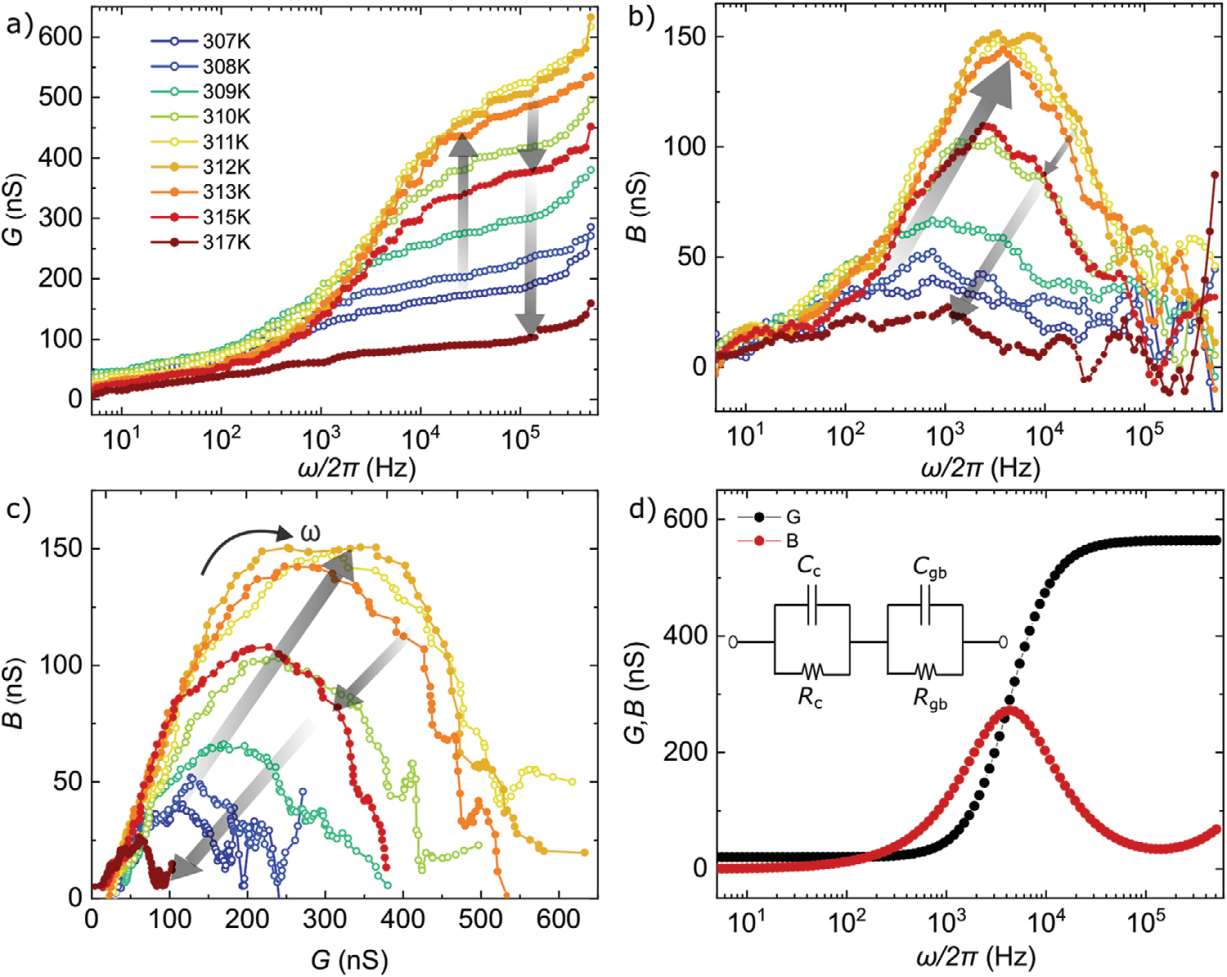
Frequency spectrum of a) the conductance *G* and b) the susceptance *B* measured at different temperatures in a **1**·2CH_3_CN crystal (sample 3). The grey arrows indicate increasing (empty dots) and decreasing (filled dots) *G* and *B* with temperature. c) Nyquist representation of the admittance. A full semicircle spans over most of the frequency range, while a small high‐frequency tail can be observed for the highest and lowest temperatures. Gray arrows indicate the shift of the curves under increasing temperature. d) Theoretical *G* and *B* curves reproducing the experimental results by using two parallel RC circuits connected in series. Each subcircuit accounts for the bulk (*C*_*c*_, *R*_*c*_) and grain boundaries (*C*_*gb*_, *R*_*gb*_) contributions.

Frequency dependent AC admittance spectroscopy has been performed at different temperatures. AC admittance spectroscopy provides a direct measurement of the different contributions to the conduction in the crystal, like grain boundaries, charge traps in bulk, etc,^[^ ^49^ ^]^ and their evolution with temperature. **Figure** **2**a,b shows *G* and *B* measured in a single crystal (sample 3) as a function of the frequency (5 Hz <*ω*/2*π* < 510 kHz) at different temperatures. At a fixed temperature, *G* increases with *ω* and begins to saturate at high frequencies (≈10^4^ Hz) and *B* develops a wide peak in the same frequency range (≈5 × 10^3^ Hz). The saturation point in *G* and the peak in *B* grow with temperature (empty dots) up to 311 K and thereafter it decays (full dots) back to the initial values, in accordance to the observations in Figure 1. Interestingly, an additional sharp increment in *B* appears at the highest frequencies (>10^5^ Hz), more clearly at higher temperatures. The full‐range frequency response can be qualitatively reproduced with two parallel RC equivalent circuits connected in series as seen in Figure 2d.

This configuration is typically used to describe proton transport as the sum of two contributions: i) the intrinsic conductance of bulk, limited by charge traps, defects, etc (*C*_*c*_ and *R*_*c*_) defining the low‐frequency spectrum. ii) The internal interfaces created by grain boundaries, etc (*C*_*gb*_ and *R*_*gb*_), defining the high‐frequency spectrum.^[^ ^49^ ^]^ Note that the frequency response is inverted with respect to the impedance. Figure 2c shows the Nyquist plot of the admittance obtained from Figures 2a,b and its temperature dependence (see equivalent Nyquist plot of the impedance in Figure S15, Supporting Information). A single, almost complete semicircle is observed spanning over most of the frequency range. This indicates that *C*_*c*_ is predominant, giving rise to the semicircle, while *C*_*gb*_ is negligible in comparison, giving rise to the high‐frequency tail observed in Figure 2c. This has been reported for negligible grain boundaries in single crystals^[^ ^43^ ^]^ and in the presence of an almost continuous path for proton transport, like a water or solvent film.^[^ ^34^ ^]^ It therefore reinforces the vehicle transport of protons through an acetonitrile liquid‐like environment scenario. The high‐frequency tail becomes significant only at the lowest and the highest temperatures where the acetonitrile film may have discontinuities and therefore the grain boundaries may start to play a role (see schematics in Figure 1a).

**Figure 3:**
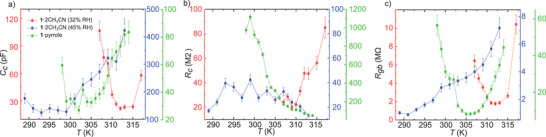
Temperature dependence of a) *C*_c_, b) *R*_c_, and c) *R*_gb_ for the **1**·2CH_3_CN at 32% RH (red dots, sample 3) and at 45% RH (blue dots, sample 4) and the pyrrole‐substituted **1**·pyrrole (green dots, sample 6), respectively. These values are obtained from the boundaries in the respective Nyquist plots, as detailed in Section S5, Supporting Information. Error bars account for the uncertainty in determining the boundaries of the semicircle due to the experimental noise.

The capacitance and resistance values of the equivalent circuit can be obtained directly from the radius and the low and high frequency boundaries of the semicircle. (See Section S5, Supporting Information, for the full mathematical description.) **Figure** **3** shows the evolution of the different components with temperature (red dots). We observe a clear drop of *C*_*c*_ that stabilizes after *T*_*c*_(aceto). In contrast, *R*_*c*_ remains approximately constant until *T*_*c*_ where it starts to increase with the temperature. On the other hand, *R*_*gb*_ decreases until *T*_*c*_(aceto) and thereafter starts to increase. Variations in the high‐frequency tail are too subtle to observe clear changes in *C*_*gb*_ with temperature (see Figure S10, Supporting Information). It seems therefore that the initial increment in *G* in Figure 1b is mainly due to a drop in *C*_*c*_ and *R*_*gb*_. The drop in *R*_*gb*_ fits with the emergence of an acetonitrile film that smooths the effect of the grain boundaries. A rapid decrease of *C*_*c*_ could be associated to a reduction of the bulk relative permittivity ϵ or the ratio *A*/*l* between the section *A* and the length *l* of the crystal. The first may be possible since acetonitrile (ϵ = 37.5) is initially being replaced by air (ϵ = 1) in the lattice. A similar effect has been previously reported for MOFs.^[^ ^34^ ^]^ The second scenario may be also possible since the crystal clearly shrinks to accommodate the loss of acetonitrile. However, a change in the dimensions would also induce an increment in *R*_*c*_ before *T*_*c*_. The latter is not observed in the experiments. Finally, the subsequent drop in *G* coincides with an increment in *R*_*c*_ and *R*_*gb*_. This again points to the appearance of domains and grain boundaries as a result of the loss of acetonitrile.

**Figure 4:**
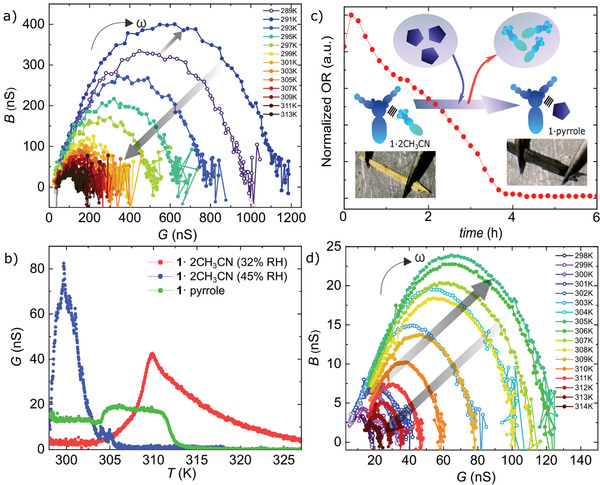
a) Nyquist representation of the admittance measured on a **1**·2CH_3_CN crystal (sample 4) at 45% RH. The presence of water shifts *T*_*c*_ to lower temperatures (≈291 K). b) *G* measured as a function of *T* in a **1** ·2CH_3_CN crystal at 45% RH, a **1** ·2CH_3_CN crystal at 32% RH (same as in Figure 1) and a **1**·pyrrole crystal (sample 6) containing pyrrole (dark green state) in the lattice. c) Optical reflectance measured on a single crystal (sample 5) by replacing acetonitrile molecules by pyrrole molecules. The color changes from a light yellow (acetonitrile in the lattice) to a dark green (pyrrole in the lattice). d) Nyquist representation of the admittance measured on a **1**·pyrrole crystal (sample 6). The semicircles do not fully close at low frequencies, indicating that *R*_*c*_ and *R*_*gb*_ are significantly larger.

To understand the role of humidity on proton transport, we have studied the AC admittance spectroscopy at higher relative humidity (RH) levels. **Figure** **4**a shows the Nyquist plot measured on a sample 4 at different temperatures and at 45 % RH. The corresponding *G* and *B* frequency spectra can be found in Supporting Information. Three main differences can be observed with respect to sample 3 (Figure 2) measured at 32% RH: i) *G* is around one order of magnitude higher at room temperature. ii) No peak is observed at *T*_*c*_(aceto) = 310 K. A single semicircle appears in the Nyquist plot that reaches a maximum at *T*_*c*_(water) = 291 K and thereafter continuously shrinks when heating the crystal. The same behaviour is observed in *G* vs *T* at a fixed frequency (see Figure 4b for a comparison [blue dots]). iii) The crystals change from yellow to orange at lower temperatures (291 K) and seem drastically modified after exposition to high humidity. Their electro‐optical properties are not reversible by adding acetonitrile again. We systematically observe this behavior in measurements where RH lies above 40%.

The larger conductance observed at room temperature could be due to additional water‐assisted proton transport. The absence of a peak at *T*_*c*_(aceto) may indicate that acetonitrile has been already displaced by the water molecules. Thus, the acetonitrile‐mediated proton transport mechanism is quenched. Density‐functional theory (DFT) calculations were performed to model **1** ·2CH_3_CN and the same material where the weakly bounded acetonitrile molecules are replaced by water at the same lattice positions (see Section S14, Supporting Information, for details). The binding energy of water and acetonitrile are found to be similar and therefore, we can expect that they may compete, especially at high humidity levels, to be adsorbed within the material. Also, the lower *T*_*c*_ observed in sample 4 may be due to the slightly lower binding energy predicted by DFT for water (0.78 eV) as compared to acetonitrile (0.85 eV). DFT predicts a H‐bonding interaction of the guest water with the BF${}_{4}^{-}$ counterions in the npCP with the OH groups pointing towards the fluorine atoms. It has been recently reported that water may displace not only acetonitrile but also further molecules in the bulk which would explain the irreversibility of the process at high humidity levels.^[^ ^45^ ^]^

The capacitance and resistance values obtained from the Nyquist plot (blue dots in Figure 3) can be compared with sample 3. In both samples, the initial *C*_*c*_ value is roughly the same (120‐150 pF). However, in contrast to sample 3, *C*_*c*_ steadily increases with temperature. This reinforces the idea that acetonitrile (ϵ = 37.5) is being replaced by water (ϵ = 80). On the other hand, *R*_*gb*_ is significantly lower than in sample 3 at room temperature and steadily increases with temperature. This indicates that, in this case, water provides the initial transport path for protons. Besides, the continuous drop of *G* involves the enhancement of grain boundaries that would in turn explain the change observed in the crystals. *R*_*c*_ remains virtually unchanged.

Finally, to delve into the role of the weakly coupled molecules in the crystal lattice, we have replaced the acetonitrile uncoordinated to the Fe ion by pyrrole. The **1**·pyrrole is obtained by exposing **1**·2CH_3_CN to a pyrrole‐saturated atmosphere (See Section S9, Supporting Information, for synthesis details). The assimilation of pyrrole by the crystal lattice and the crystallinity of the new material is shown by thermogravimetric analysis, infrared spectroscopy, and powder X‐ray diffraction, see Sections S10–S13, Supporting Information. Figure 4c shows the optical reflectance and optical images of a crystal (sample 5) before and after exposition to a pyrrole‐saturated atmosphere at room temperature. The crystal color changes from light yellow to a dark green, indicating a modification of the crystal lattice (see supporting video).

Figure 4b shows *G* measured as a function of *T* and at *ω*/2*π* = 10 kHz on a **1**·pyrrole crystal (sample 5) soaked with pyrrole (dark green state, green dots) and at 33% RH (see *B* and additional examples in Figure S13, Supporting Information). *G* is non‐zero at room temperature, in contrast with sample 1 containing acetonitrile (red dots). In addition, a sharp increment appears at *T*_*c*_(pyrr) = 305 K and *G* remains roughly constant until 312 K where it rapidly decreases below the initial, room temperature *G*. The decay temperature (≈ 312 K) is similar to the one observed for **1**·2CH_3_CN crystals and the plateau can be roughly fitted by two contributions that tentatively can account for the initial loss of pyrrole (305 K) and a subsequent loss of remaining acetonitrile (310 K). The presence of both molecules in the lattice seems in accordance to the DFT calculations. The calculations could not converge to a fully relaxed geometry when all weakly bounded acetonitrile molecules were replaced by pyrrole. Among the different studied configurations of the pyrrole inside the material, the lowest energy calculation yields a structure with pyrrole bound in a *π* − *π* stacking with the pyrazine ligand. This would explain the appearance of a plateau or double peak in the conductance.

A similar temperature dependence is observed in the Nyquist representation of the admittance, as shown in Figure 4d (see Figure S12, Supporting Information, for the corresponding *G* and *B* frequency spectra). The overall conductance is significantly smaller than in the previous samples. Accordingly, the semicircle shifts to lower frequencies and appears incomplete. Besides, by checking in detail the equivalent circuit parameters (green dots in Figure 3), *R*_*gb*_ and *C*_*c*_ seems to be following the same pattern than sample 3 but shifted to lower temperatures. This points to a similar mechanism for the conductance. This is more clearly seen in the normalized figures shown in Figure S11, Supporting Information. In contrast, *R*_*c*_ is much larger and seems to follow a different trend. The ability of the crystal to selectively accommodate different molecules in the lattice thus determines the optical properties (color) as well as proton transport properties like conductance or transition temperature.

## 3 Conclusion

In conclusion, we have studied the charge transport properties of **1**·2CH_3_CN nonporous coordination polymers acting as porous by means of AC admittance spectroscopy. We show that the conductance in the crystals is of protonic origin. A vehicle transport mechanism is triggered by the dynamics of weakly coupled acetonitrile molecules in the lattice, that leads to a non‐trivial modification of the capacitance and resistance of the material. A proper combination of thermal cycles allows to activate and maintain a current level on the otherwise insulating material. Acetonitrile therefore provides **1**·2CH_3_CN with proton transport capabilities even at low humidity levels. Besides, we have in situ generated the new **1**·pyrrole material. We find that proton conductivity and optical response (color) can be tuned thanks to the ability of the crystals to reorganize when different molecules are incorporated in the lattice. Our findings may serve as starting point to the design of switchable protonic conductors and capacitive sensors with selectivity to different small molecules and at low humidity levels.

## 4 Experimental Section

### Materials

Chemicals and reagents were purchased from commercial suppliers and used as received

### Optcal Reflectivity

Optical reflectivity measurements between and 373 K were performed using a MOTIC SMZ‐171 optical stereoscope coupled with a MOTICAM 3. Images were collected in BMP format without any filter using the Motic Images Plus 3.0 software, with the mean value from each region of interest (ROI) analyzed under the ImageJ program. The temperature was controlled using a Linkam T95 system controller and a LNP 95 Liquid Nitrogen Cooling System.

### Thermogravimetric Analysis

TGA was performed using a TA Instrument TGAQ500 with a ramp of 1 °C min^−1^ under air from 303 to 873 K.

### Infrared Spectroscopy

FT‐IR spectra were recorded as neat samples in the range 400–4000 cm^−1^ on a Bruker Tensor 27 (ATR device) Spectrometer.

### Elemental analysis

Elemental analyses (C, H, and N) were performed on a LECO CHNS‐932 Analyzer at the “Servicio Interdepartamental de Investigación (SIdI)” at Autónoma University of Madrid

### X‐ray Diffraction Spectroscopy

Powder X ray diffraction data was collected in a Rigaku Smartlab SE diffractometer with a Bragg‐Brentano configuration, using Cu‐K*α* radiation (*λ* = 0.1541 nm). The sample was measured between 5° and 50° with a speed of 1.8° min^‐1^ under an X‐Ray fluorescence reduction mode, at room temperature.

### AC Admittance Spectroscopy

Temperature‐dependent AC spectroscopy is performed in a Linkam T95 electrical probe station equipped with a LNP 95 liquid nitrogen cooling system and a Zurich Instruments lock‐in amplifier.

### Density Functional Theory Calculations

The full geometrical optimization of the unit cell of 1, 1.2CH_3_CN and 1.2H_2_O compounds are performed using the QuantumESPRESSO package.^[^ ^50^, ^51^ ^]^

## Conflict of Interest

The authors declare no conflict of interest.

## Supporting information

Supporting Information

Supplemental Movie 1

## Data Availability

The data that support the findings of this study are available from the corresponding author upon reasonable request.

## References

[1] J. Sánchez Costa , S. Rodríguez‐Jiménez , G. A. Craig , B. Barth , C. M. Beavers , S. J. Teat , G. Aromí , J. Am. Chem. Soc. 2014, 136, 3869. 24555786 10.1021/ja411595y

[2] J. S. Costa , S. Rodríguez‐Jiménez , G. A. Craig , B. Barth , C. M. Beavers , S. J. Teat , K. J. Gagnon , L. A. Barrios , O. Roubeau , G. Aromí , Inorg. Chem. Front. 2020, 7, 3165.

[3] E. Coronado , M. Giménez‐Marqués , G. M. Espallargas , L. Brammer , Nat. Commun. 2012, 3, 828. 22569372 10.1038/ncomms1827

[4] J. Miguel‐Donet , J. López‐Cabrelles , N. Calvo Galve , E. Coronado , G. Mínguez Espallargas , Chem. ‐ A Eur. J. 2018, 24, 12426. 10.1002/chem.201802510 29989253

[5] A. Thirumurugan , W. Li , A. K. Cheetham , Dalt. Trans. 2012, 41, 4126. 10.1039/c2dt12330d 22378230

[6] C. Yu , M. G. Cowan , R. D. Noble , W. Zhang , Chem. Commun. 2014, 50, 5745. 10.1039/c4cc02143f 24752375

[7] A. Tarassoli , V. Nobakht , E. Baladi , L. Carlucci , D. M. Proserpio , CrystEngComm 2017, 19, 6116.

[8] B. Xiao , P. J. Byrne , P. S. Wheatley , D. S. Wragg , X. Zhao , A. J. Fletcher , K. M. Thomas , L. Peters , J. S. Evans , J. E. Warren , W. Zhou , R. E. Morris , Nat. Chem. 2009, 1, 289. 21495253 10.1038/nchem.254

[9] E. Fernandez‐Bartolome , E. Resines‐Urien , M. Murillo‐Vidal , L. Piñeiro‐Lopez , J. Sánchez Costa , Inorg. Chem. Front. 2021, 8, 2426.

[10] E. Resines‐Urien , L. Piñeiro‐López , E. Fernandez‐Bartolome , A. Gamonal , M. Garcia‐Hernandez , J. Sánchez Costa , Dalt. Trans. 2020, 49, 7315. 10.1039/d0dt01533d 32469360

[11] S. Rodríguez‐Jiménez , H. L. Feltham , S. Brooker , Angew. Chemie ‐ Int. Ed. 2016, 55, 15067. 10.1002/anie.201608813 27730720

[12] S. Jeoung , S. H. Sahgong , J. H. Kim , S. M. Hwang , Y. Kim , H. R. Moon , J. Mater. Chem. A 2016, 4, 13468.

[13] U. S. Arrozi , V. Bon , C. Kutzscher , I. Senkovska , S. Kaskel , Dalt. Trans. 2019, 48, 3415. 10.1039/c8dt03866j 30788474

[14] X. Su , L. Bromberg , V. Martis , F. Simeon , A. Huq , T. A. Hatton , ACS Appl. Mater. Interfaces 2017, 9, 11299. 28244732 10.1021/acsami.7b02471

[15] C.‐T. Yang , A. R. Kshirsagar , A. C. Eddin , L.‐C. Lin , R. Poloni , Chem. ‐ A Eur. J. 2018, 24, 15167. 10.1002/chem.201804014 30110512

[16] J. Li , X. Wang , G. Zhao , C. Chen , Z. Chai , A. Alsaedi , T. Hayat , X. Wang , Chem. Soc. Rev. 2018, 47, 2322. 29498381 10.1039/c7cs00543a

[17] A. Gamonal , C. Sun , A. L. Mariano , E. Fernandez‐Bartolome , E. Guerrero‐SanVicente , B. Vlaisavljevich , J. Castells‐Gil , C. Marti‐Gastaldo , R. Poloni , R. Wannemacher , J. Cabanillas‐Gonzalez , J. S. Costa , J. Phys. Chem. Lett. 2020, 11, 3362. 32195588 10.1021/acs.jpclett.0c00457

[18] V. Rubio‐Giménez , S. Tatay , C. Martí‐Gastaldo , Chem. Soc. Rev. 2020, 49, 5601. 32643717 10.1039/c9cs00594c

[19] L. Sun , S. S. Park , D. Sheberla , M. Dincǎ , J. Am. Chem. Soc. 2016, 138, 14772. 27766856 10.1021/jacs.6b09345

[20] L. Sun , M. G. Campbell , M. Dincǎ , Angew. Chemie Int. Ed. 2016, 55, 3566. 10.1002/anie.201506219 26749063

[21] D. Sheberla , J. C. Bachman , J. S. Elias , C. J. Sun , Y. Shao‐Horn , M. Dincǎ , Nat. Mater. 2017, 16, 220. 27723738 10.1038/nmat4766

[22] H. Ding , W. Wu , C. Jiang , Y. Ding , W. Bian , B. Hu , P. Singh , C. J. Orme , L. Wang , Y. Zhang , D. Ding , Nat. Commun. 2020, 11, 1907. 32312963 10.1038/s41467-020-15677-z PMC7171140

[23] J. F. Nagle , H. J. Morowitz , Proc. Natl. Acad. Sci. U. S. A. 1978, 75, 298. 272644 10.1073/pnas.75.1.298 PMC411234

[24] M. Yoon , K. Suh , S. Natarajan , K. Kim , Angew. Chemie ‐ Int. Ed. 2013, 52, 2688. 10.1002/anie.201206410 23345157

[25] D.‐W. Lim , H. Kitagawa , Chem. Rev. 2020, 120, 8416. 32407101 10.1021/acs.chemrev.9b00842

[26] E. Pardo , C. Train , G. Gontard , K. Boubekeur , O. Fabelo , H. Liu , B. Dkhil , F. Lloret , K. Nakagawa , H. Tokoro , S. I. Ohkoshi , M. Verdaguer , J. Am. Chem. Soc. 2011, 133, 15328. 21913689 10.1021/ja206917z

[27] H. Xu , S. Tao , D. Jiang , Nat. Mater. 2016, 15, 722. 27043780 10.1038/nmat4611

[28] Y. Ye , W. Guo , L. Wang , Z. Li , Z. Song , J. Chen , Z. Zhang , S. Xiang , B. Chen , J. Am. Chem. Soc. 2017, 139, 15604. 29072912 10.1021/jacs.7b09163

[29] Y.‐S. Wei , X.‐P. Hu , Z. Han , X.‐Y. Dong , S.‐Q. Zang , T. C. W. Mak , J. Am. Chem. Soc. 2017, 139, 3505. 28192991 10.1021/jacs.6b12847

[30] K. Müller , J. Helfferich , F. Zhao , R. Verma , A. B. Kanj , V. Meded , D. Bléger , W. Wenzel , L. Heinke , Adv. Mater. 2018, 30, 1706551. 10.1002/adma.201706551 29315923

[31] S.‐S. Liu , Z. Han , J.‐S. Yang , S.‐Z. Huang , X.‐Y. Dong , S.‐Q. Zang , Inorg. Chem. 2019, 59, 396. 31851507 10.1021/acs.inorgchem.9b02649

[32] W.‐L. Xue , W.‐H. Deng , H. Chen , R.‐H. Liu , J. M. Taylor , Y.‐k. Li , L. Wang , Y.‐H. Deng , W.‐H. Li , Y.‐Y. Wen , G.‐E. Wang , C.‐Q. Wan , G. Xu , Angew. Chemie Int. Ed. 2021, 60, 1290. 10.1002/anie.202010783 32996683

[33] P. Rought , C. Marsh , S. Pili , I. P. Silverwood , V. G. Sakai , M. Li , M. S. Brown , S. P. Argent , I. Vitorica‐Yrezabal , G. Whitehead , M. R. Warren , S. Yang , M. Schröder , Chem. Sci. 2019, 10, 1492. 30809366 10.1039/c8sc03022g PMC6354967

[34] P. Barbosa , N. C. Rosero‐Navarro , F. N. Shi , F. M. Figueiredo , Electrochim. Acta 2015, 153, 19.

[35] H. Zhang , Z. A. Yan , Z. M. Wu , Z. Q. Lin , W. M. Liao , J. He , J. Solid State Chem. 2020, 287, 121325.

[36] S. Wang , M. Wahiduzzaman , L. Davis , A. Tissot , W. Shepard , J. Marrot , C. Martineau‐Corcos , D. Hamdane , G. Maurin , S. Devautour‐Vinot , C. Serre , Nat. Commun. 2018, 9, 4937. 30467390 10.1038/s41467-018-07414-4 PMC6250719

[37] K. Zhang , X. Xie , H. Li , J. Gao , L. Nie , Y. Pan , J. Xie , D. Tian , W. Liu , Q. Fan , Adv. Mater. 2017, 29, 1701804. 10.1002/adma.201701804 28681956

[38] A. Shigematsu , T. Yamada , H. Kitagawa , J. Am. Chem. Soc. 2011, 133, 2034. 21284399 10.1021/ja109810w

[39] P. Ramaswamy , R. Matsuda , W. Kosaka , G. Akiyama , H. Joon Jeon , S. Kitagawa , Chem. Commun. 2014, 50, 1144. 10.1039/c3cc47980c 24322717

[40] D. Umeyama , S. Horike , M. Inukai , T. Itakura , S. Kitagawa , J. Am. Chem. Soc. 2012, 134, 12780. 22783808 10.1021/ja304693r

[41] S. Pili , S. P. Argent , C. G. Morris , P. Rought , V. García‐Sakai , I. P. Silverwood , T. L. Easun , M. Li , M. R. Warren , C. A. Murray , C. C. Tang , S. Yang , M. Schröder , J. Am. Chem. Soc. 2016, 138, 6352. 27182787 10.1021/jacs.6b02194 PMC4882730

[42] H. Liu , R. Li , J. Lu , Z. Liu , S. Wang , H. Tian , Cryst. Eng. Comm. 2020, 22, 6935.

[43] J. Stankiewicz , M. Tomás , I. T. Dobrinovitch , E. Forcén‐Vázquez , L. R. Falvello , Chem. Mater. 2014, 26, 5282.

[44] L. Feng , Z.‐Q. Pan , H. Zhou , M. Zhou , H.‐B. Hou , Dalt. Trans. 2019, 48, 16493. 10.1039/c9dt02960e 31497810

[45] R. F. Mendes , P. Barbosa , E. M. Domingues , P. Silva , F. Figueiredo , F. A. A. Paz , Chem. Sci. 2020, 11, 6305. 32874517 10.1039/d0sc01762k PMC7448532

[46] S. Horike , D. Umeyama , S. Kitagawa , Acc. Chem. Res. 2013, 46, 2376. 23730917 10.1021/ar300291s

[47] E. Resines‐Urien , E. Burzurí , E. Fernandez‐Bartolome , M. Á. García García‐Tuñón , P. De La Presa , R. Poloni , S. J. Teat , J. S. Costa , Chem. Sci. 2019, 10, 6612. 31367312 10.1039/c9sc02522g PMC6625413

[48] N. S. Gould , S. Li , H. J. Cho , H. Landfield , S. Caratzoulas , D. Vlachos , P. Bai , B. Xu , Nat. Commun. 2020, 11, 1060. 32103007 10.1038/s41467-020-14860-6 PMC7044222

[49] P. R. Bueno , J. A. Varela , E. Longo , J. Eur. Ceram. Soc. 2007, 27, 4313.

[50] P. Giannozzi , S. Baroni , N. Bonini , M. Calandra , R. Car , C. Cavazzoni , D. Ceresoli , G. L. Chiarotti , M. Cococcioni , I. Dabo , A. Dal Corso , S. De Gironcoli , S. Fabris , G. Fratesi , R. Gebauer , U. Gerstmann , C. Gougoussis , A. Kokalj , M. Lazzeri , L. Martin‐Samos , N. Marzari , F. Mauri , R. Mazzarello , S. Paolini , A. Pasquarello , L. Paulatto , C. Sbraccia , S. Scandolo , G. Sclauzero , A. P. Seitsonen , et al., J. Phys. Condens. Matter 2009, 21, 395502. 21832390 10.1088/0953-8984/21/39/395502

[51] P. Giannozzi , O. Andreussi , T. Brumme , O. Bunau , M. B. Nardelli , M. Calandra , R. Car , C. Cavazzoni , D. Ceresoli , M. Cococcioni , N. Colonna , I. Carnimeo , A. D. Corso , S. de Gironcoli , P. Delugas , R. A. DiStasio , A. Ferretti , A. Floris , G. Fratesi , G. Fugallo , R. Gebauer , U. Gerstmann , F. Giustino , T. Gorni , J. Jia , M. Kawamura , H.‐Y. Ko , A. Kokalj , E. Küçükbenli , et al., J. Phys. Condens. Matter 2017, 29, 465901. 29064822 10.1088/1361-648X/aa8f79

