# Supplementary material for: Tunable Proton Conductivity and Color in a Nonporous Coordination Polymer via Lattice Accommodation to Small Molecules
Source: Adv Sci (Weinh). 2021 Oct 18;8(22):2102619. doi: 10.1002/advs.202102619 (PMC8596141; doi:10.1002/advs.202102619)
Supplement: Supplementary file 1 — Supporting Information [file ADVS-8-2102619-s001.pdf]

© 2021 Wiley-VCH GmbH

ADVANCED SCIENCE
Open Access

Supporting Information

for *Adv. Sci.,* DOI: 10.1002/advs.202102619

Tunable Proton Conductivity and Color in a Nonporous Coordination Polymer via Lattice Accommodation to Small Molecules

Aysegul Develioglu, Esther Resines-Urien, Roberta Poloni, Lucía Martín-Pérez, Jose Sanchez Costa, and Enrique Burzurí**

Supporting Information of

Tunable Proton Conductivity and Color in a Nonporous Coordination Polymer via Lattice Accommodation to Small Molecules.

Aysegul Develioglu[1], Esther Resines-Urien[1], Roberta Poloni[2], Lucía Martín-Pérez[1], Jose Sanchez Costa[1,*] and Enrique Burzurí[1,*]

[1]IMDEA Nanociencia, Campus de Cantoblanco, Calle Faraday 9, 28049 Madrid, Spain
[2]Université Grenoble Alpes, CNRS, SIMAP, Grenoble, 38000, France

Contents

1. Crystal structure of 1·2CH$_3$CN 1
2. Additional admittance measurements as a function of the temperature 3
3. Statistics 6
4. Additional admittance measurements as a function of time 7
5. Mathematical description of the electronic equivalent circuit 7
6. Additional representation of R and C as a function of temperature. 10
7. Frequency spectrum of G and B of pyrrole-substituted crystal 11
8. Time constant , equivalent impedance Nyquist plots and comparative of the conductance of the materials 13
9. Synthesis of $_\infty${[Fe(H$_2$O)$_2$(CH$_3$CN)$_2$(pyrazine)](BF$_4$)$_2$·(CH$_3$CN)$_2$} (1·2CH$_3$CN) and $_\infty${[Fe(H$_2$O)$_2$(CH$_3$CN)$_2$(pyrazine)](BF$_4$)$_2$·pyrrole} (1·pyrrole) 14
10. Thermogravimetric analyses 15
11. Optical Reflectivity upon pyrrole exposure 17
12. Fourier Transform Infrared Spectroscopy (FTIR) 18
13. Powder X-ray Diffraction (PXRD) study 19
14. Density functional theory calculations 19
15. Bibliography 20

1. Crystal structure of 1·2CH_3CN

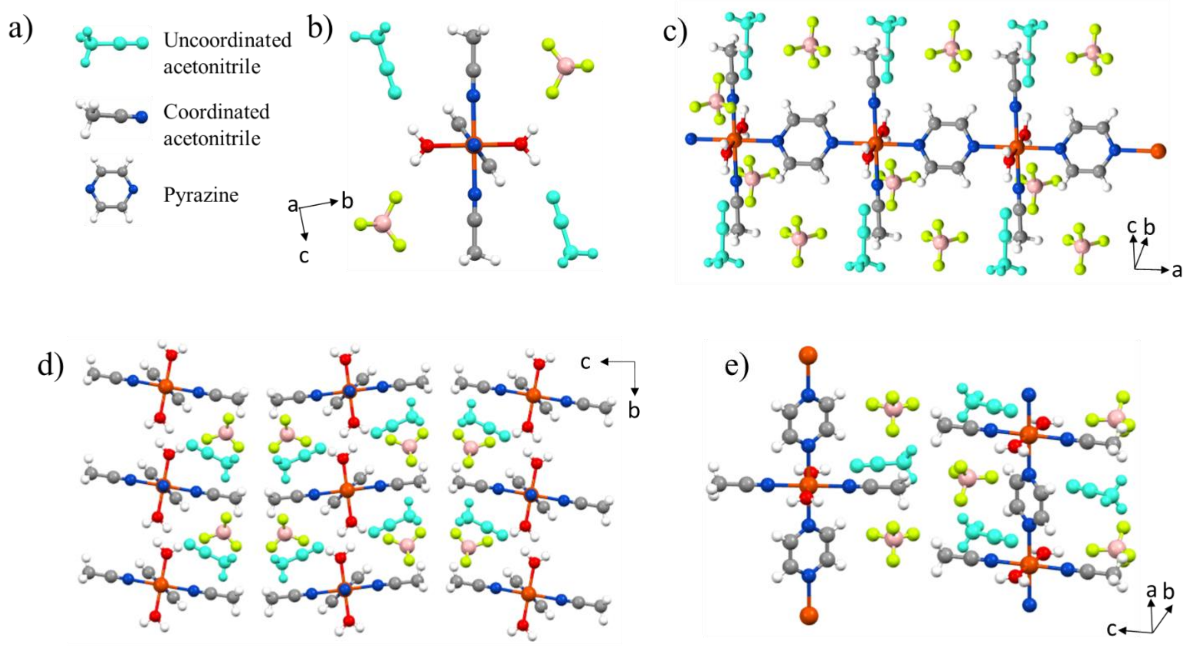

Figure S 1. Crystal structure of the 1·2CH_3CN crystal showing the position of the uncoordinated acetonitrile molecules

Table S1. Crystallographic data of **1**·$2CH_3CN$.[1]

Compound	**1**·$2CH_3CN$
CCDC	1895056
Chemical formula	$C_{12}H_{20}B_2F_8FeN_6O_2$
Formula mass	510.11 g/mol
Temperature (K)	250.0
Crystal system	Orthorhombic
Space group	*Cmca*
a/Å	7.2430(17)
b/Å	12.882(2)
c/Å	23.908(6)
α/◦	90
β/◦	90
γ/◦	90
V(Å^3)	2230.8(9)
Z	4
Radiation type	Synchrotron
Density (calculated mg m^{-3})	1.518
Absorption coefficient (mm^{-1})	0.76
F(000)	1032.0
Crystal size (mm^3)	0.000576
Goodness of fit on F^2	1.112
R1, wR2 [I>2σ(I)]	0.0719, 0.2069
R1, wR2 (all data)	0.0930, 0.2330

Often[2,3] the porosity of a sample is measured by means of adsorption isotherms, this procedure involves the prior degassing of the material, by vacuum and/or the application of temperature. In our case, this results in a loss of the non-coordinated acetonitrile molecule in the structure, and is therefore not a suitable option.

Nevertheless, several close-related works[4–6] justify the absence of pores by showing the structure in the spacefill mode (covalent ratii), where the presence of any pores, if present, should be visible. In our case, the view of the 1·$2CH_3CN$ coordination polymer illustrates that the polymer is tightly packed, with no visible pores or voids in the structure, as shown in Figure S2.

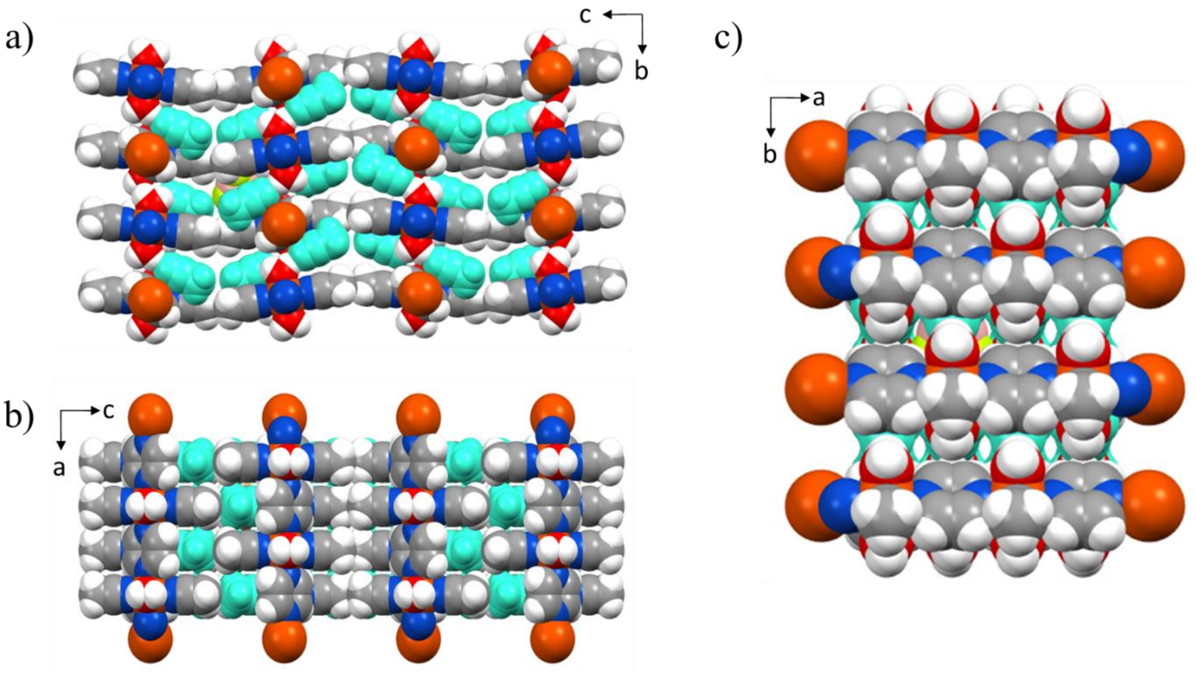

Figure S 2 View of 1·2CH_3CN in the spacefill mode (covalent ratii) along the a) a axis, b) b axis and c) c axis.

Another widely used technique[7–9] to support the absence of porosity in a material is the use of the PLATON software to calculate the voids in the structure. We have follow this procedure for 1·2CH_3CN, and there are no residual voids accessible by the solvent, irrespective of whether it is calculated with the CALC VOID or SQUEEZE functions. The default Van der Waals radius has been used (1.2 Å).[10]

To further address the non-porosity of the material, the voids have also been calculated with the Olex2 program.[11]. For 1·2CH_3CN the result is the same as with PLATON, that there is no accessible volume for solvent or guest molecules.

There are multiple reports of molecule exchange in non-porous coordination polymers, where the exchange takes place through a process of diffusion throughout the crystal lattice.[4,5,12] The diffusion of the molecules induces a reorganization of the crystal that quenches the potential voids that can be created along the process.[13] We propose that this same type of process is what occurs in 1·2CH_3CN.

2. Additional admittance measurements as a function of the temperature

Figure S3-5 show additional measurements of the temperature dependence of the conductance *G* and the susceptance *B* measured in three different **1**·2CH_3CN crystals with a lock-in technique. The AC excitation is set to V_{AC} = 100 mV and $\omega/2\pi$ = 510 kHz. A 1 V DC offset is applied. In this frequency, *G* shows a reproducible sharp peak at T_c as observed in Figure 1 of the main manuscript. In contrast, *B* shows an oscillatory behaviour as a consequence of the non-trivial evolution of the high-frequency tail, associated to the grain boundaries, with the temperature (see main text).

The initial increment in temperature can be fitted with an Arrhenius law for activated transport (Figures S3-S5(c)).

$$\boldsymbol{\tau = \tau_0 exp\left(-\frac{E_a}{k_B T}\right)} \qquad \textbf{Eq. 1}$$

where τ_0 is the pre-exponential factor, E_a is the activation energy, k_B is the Boltzmann constant. The activation energy barriers E_a obtained from the fit are 2.08 eV, 2.75 eV and 2.02 eV, for the three respective figures. These values are relatively high when compared with other reports for vehicle proton transport in coordination polymers.

The red lines in Figures S3-S5(a) are fits to the exponential decays after T_c to the formula:

$$\boldsymbol{\tau_{III}(\mathrm{K}) = \tau_{0,\mathrm{K}} \mathrm{exp}\left(-\frac{\mathrm{T}}{\tau_\mathrm{K}}\right) + \mathrm{A}} \qquad \textbf{Eq. 2}$$

where $\tau_{0,\mathrm{K}}$ is the pre-exponential factor, τ_K is the decay rate in temperature, and A accounts for temperature-independent contributions to $\boldsymbol{\tau}$ .The decay rates in kelvin obtained from Figures S3-S5 are τ_K = 2.00 K, 1.75 K and 2.04 K, respectively.

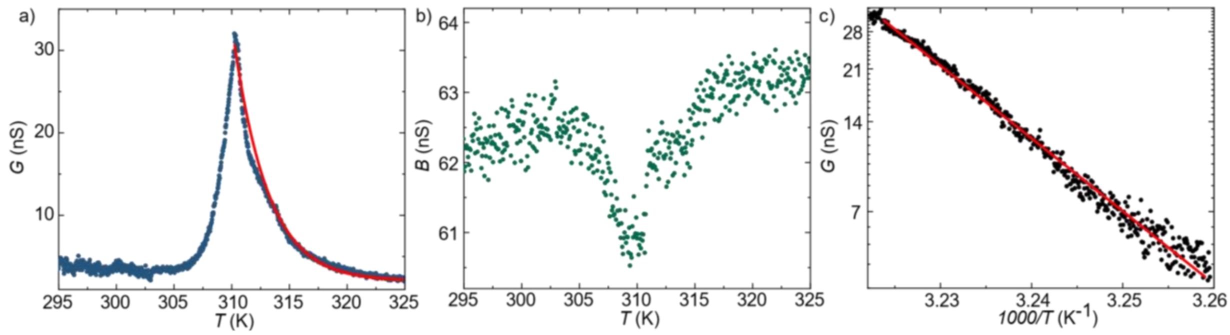

Figure S 3. (a) Conductance *G* and (b) susceptance *B* measured as a function of the temperature *T* with a lock-in technique. A peak appears at T_c(aceto) = 310 K. The solid line in (a) is a fit to Eq.2 with τ_K = 2.00 K. (c) Arrhenius plot of *G* below T_c(aceto). The solid line is a fit to Eq. 1 with E_a = 2.08 eV.

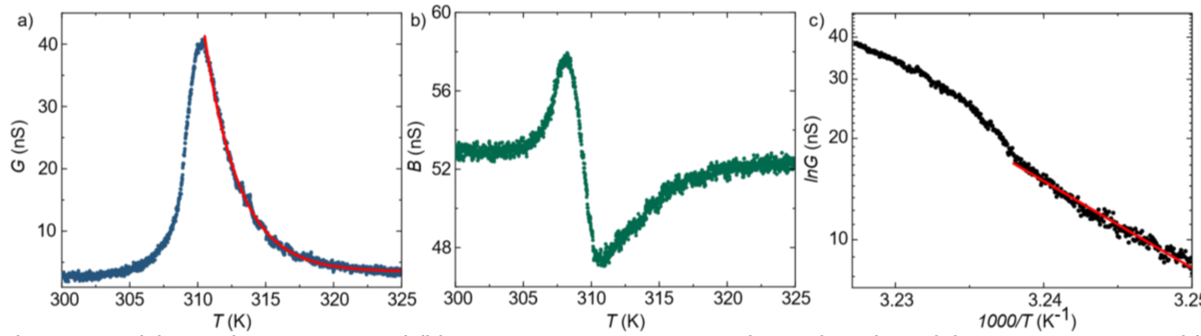

Figure S 4. (a) Conductance G and (b) susceptance B measured as a function of the temperature T with a lock-in technique. A peak appears at T_c(aceto) = 310 K. The solid line in (a) is a fit to Eq.2 with $\boldsymbol{\tau_K}$ = 1.75 K. (c) Arrhenius plot of G below T_c(aceto). The solid line is a fit to Eq. 1 with E_a = 2.75 eV.

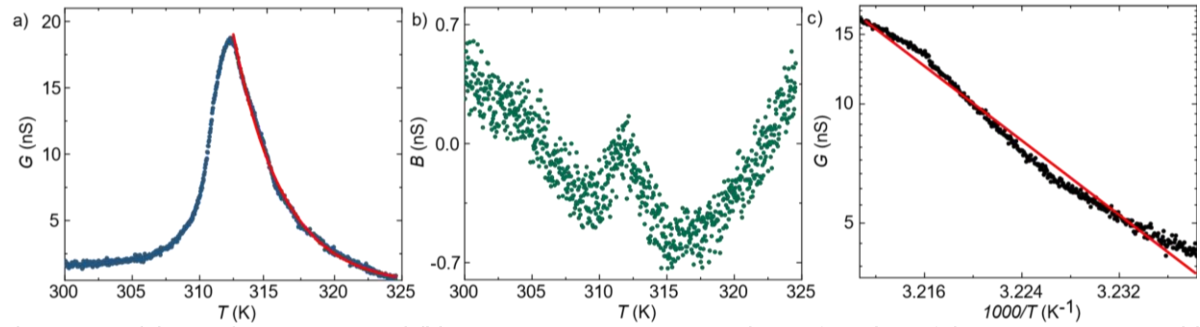

Figure S 5. (a) Conductance G and (b) susceptance B measured as a function of the temperature T with a lock-in technique. A peak appears at T_c(aceto) = 312 K. The solid line in (a) is a fit to Eq.2 with $\boldsymbol{\tau_K}$ = 2.04 K. (c) Arrhenius plot of G below T_c(aceto). The solid line is a fit to Eq. 1 with E_a = 2.02 eV.

Figure S6 shows G measured in two consecutive thermal cycles (heating-cooling) in a same **1**·2CH_3CN crystal. A peak appears in G only during the first heating of the sample, as reported before[1]. Therefore, proton conductance is intrinsically connected to the transient presence of free acetonitrile.

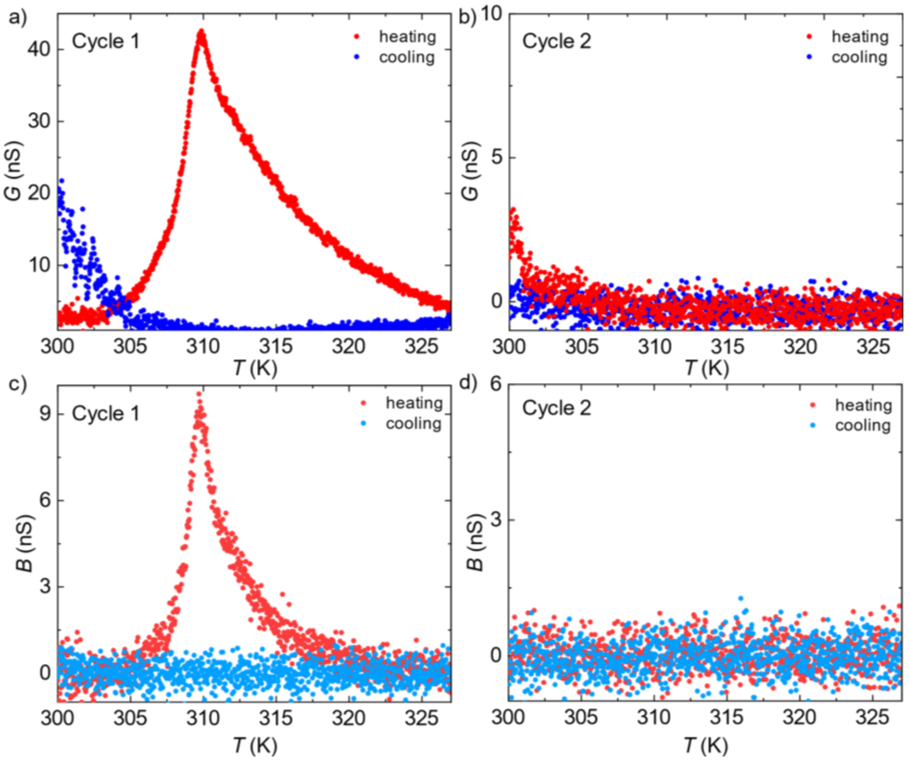

Figure S 6. *G* and *B* measured during (a,c) a first and (b,d) a second thermal (heating-cooling) cycle. The peak in *G* and *B* is only observed during the first heating cycle.

3. Statistical analysis

3.1. AC admittance spectroscopy on 1·2CH_3CN crystals under low humidity levels.

G and *B* were measured with a lock-in technique in 20 **1**·2CH_3CN crystals as a function of temperature and at humidity levels below 40% RH. A peak appears at T_c (aceto) = 310 K in 16 of them (80%). The average activation energy barrier obtained from the Arrhenius fit is 1.62 ± 0.04 eV, consistent with the vehicle transport mechanism. The average decay (τ_K) calculated from 10 samples using Eq. 2 is 3.00 ± K. The error is obtained as the standard deviation from the average.

Frequency dependent measurements were carried out in 7 crystals showing similar Nyquist representations.

3.2. AC admittance spectroscopy on 1·2CH_3CN crystals under high humidity levels (> 40%)

Frequency dependent measurements of *G* and *B* were carried out in 6 **1**·2CH_3CN crystals under humidity levels higher than 40%. The Nyquist representation is qualitatively reproducible in all the samples. G and B is measured as a function of temperature in one crystal.

3.3. AC admittance spectroscopy on 1·pyrrole crystals

G and *B* were measured as a function of temperature in 6 pyrrole-substituted **1**·pyrrole crystals. All 6 samples have non-zero *G* at RT and a wider peak shifted to lower temperatures T_c (pyrr) = 305 K (see Figure 4 in the main manuscript).

4. Additional admittance measurements as a function of time

Figure S7 shows an additional measurement of *G* as a function of time to better understand the role of time and temperature in the post-transition relaxation. The temperature is swept from room *T* up to T_c (aceto).There, *T* is kept constant and G is measured as a function of time. Note that, in contrast to the measurement shown in Figure 1 of the main manuscript, *T* is not swept back down to room *T*. Initially *G* continues increasing as seen in the main manuscript. Thereafter, no significant decay of *G* is observed in timescales comparable to those employed in the temperature-dependent measurement. The dynamics are therefore predominantly governed by the increment in temperature and not time.

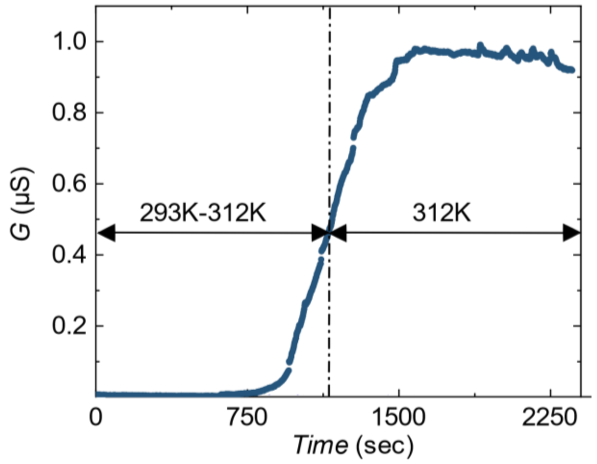

Figure S 7. *G* measured as a function of time. Initially, *T* is swept until T_c (aceto). Thereafter, *T* is kept constant and *G* is measured as a function of time.

5. Mathematical description of the electronic equivalent circuit

The frequency response of the admittance *Y** observed in the main text can be approximately reproduced with a set of two parallel RC circuits connected in series. Each sub-circuit describes the contribution to charge transport of the grain boundaries (C_{gb}, R_{gb}) and the bulk crystal (C_c, R_c)[14].

Here below, the mathematical description of the resulting circuit is explained in detail. The complex admittance *Y** (*ω*) of a circuit as a function of *ω* angular frequency can be written as the sum of conductance (*G*) and susceptance (*B*):

$$Y^{*}\,(\omega) = G(\omega) + i\,B(\omega) \tag{Eq. 3}$$

The complex admittance of a resistive and a capacitive element are respectively:

$$Y^{*}(\omega) = 1/R \tag{Eq. 4}$$

$$Y^{*}(\omega) = i\,\omega\,C \tag{Eq. 5}$$

The complex admittance of the resulting parallel RC circuit is:

$$Y^{*}\,(\omega) = \frac{1}{R} + i\,\omega\,C \tag{Eq. 6}$$

The addition of the two circuits in series would result in the total *Y** of the circuit:

$$Y^{*}\,(\omega) = \frac{1}{\frac{1}{\frac{1}{R_C}+i\,\omega\,C_C}+\frac{1}{\frac{1}{R_{gb}}+i\,\omega\,C_{gb}}} \tag{Eq. 7}$$

From Eq. 7 we can obtain the *G* and *B* values of the circuit as:

$$G = \frac{1+\omega^2 R_e(C_c^2 R_c + C_{gb}^2 R_{gb})}{(R_c+R_{gb})(1+\omega^2 R_e^2 (C_{gb}+C_c)^2)} \tag{Eq. 8}$$

$$B = \frac{\omega(C_{gb}R_{gb}^2 + C_c R_c^2) + \omega^3 C_c C_{gb} R_e^2 (C_c+C_{gb})(R_c+R_{gb})^2}{(R_c+R_{gb})^2(1+\omega^2 R_e^2 (C_c+C_{gb})^2)} \tag{Eq. 9}$$

where R_e is the equivalent resistance of the circuit:

$$R_e = \frac{R_c R_{gb}}{R_c+R_{gb}} \tag{Eq. 10}$$

The experimental B and G curves as a function of ω can be qualitatively reproduced by selecting an appropriate set of R and C parameters. Figure 2(d) in the main text is calculated by using Eq. 8 and Eq. 9.

The Nyquist diagram of Y^* is a parametric plot of B vs G using frequency as common sweeping parameter. For simple circuits, it takes the form of a semicircle, a straight line or the combination of both of them. In the particular circuit described in this manuscript, according to Eq. 8 and 9, it takes the shape shown in Figure S8.

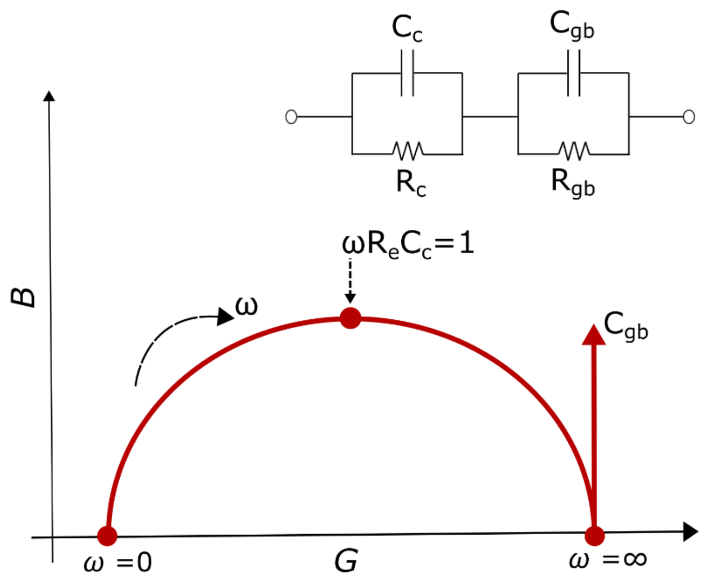

Figure S 8. Nyquist representation of the admittance Y^* of the circuit shown in the inset. The low frequency semicircle accounts for one of the RC sub-circuits, typically the one with higher C. The high-frequency tail accounts for the effect of the lower C.

Eqs. 8 and 9 can be further simplified in the low frequency ($\omega \rightarrow 0$) and the high frequency limits ($\omega \rightarrow \infty$), to obtain the boundary points of the semicircle:

$$\boldsymbol{\omega \rightarrow 0 : \left(\frac{1}{R_c+R_{gb}}, 0\right)} \qquad \textbf{Eq. 11}$$

$$\boldsymbol{\omega \rightarrow \infty : \left(\frac{1}{R_{gb}}, 0\right)} \qquad \textbf{Eq. 12}$$

These expressions are valid when C_{gb}<<C_c. R_c and R_{gb} can be therefore obtained from the boundaries of the semicircle. The largest capacitance, in this case C_c, can be obtained from the central part of the circumference that corresponds to the time constant $\tau = R_eC_c$ of the circuit.

Figure S9 shows (a) the experimental Nyquist plot shown in Figure 2 of the main manuscript and (b) the theoretical curves calculated with Eqs. 8 and 9 with the parameters shown in Figure 3 and obtained with Eqs. 11 and 12. The curves can be qualitatively well

reproduced. Small differences arise between theory and experiment. First, the experimental semicircle is flattened. This is known to be originated by the coexistence of several relaxation processes that in turn originate a distribution of relaxation times (τ) in the crystal. Second, the experimental high-frequency tail presents a finite slope whereas the theoretical calculation diverges. This is most likely due to the presence of additional *R*, *C* components not captured by the simple RC circuit described in Figure S7.

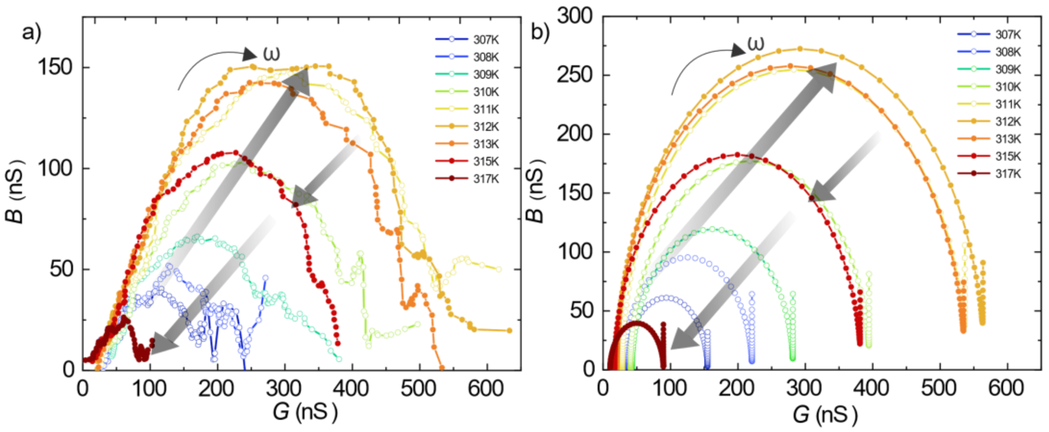

Figure S 9 (a) Experimental Nyquist representation of *Y** from Figure 2 in the main manuscript and (b) theoretical Nyquist representation of 2 parallel RC circuits connected in series, calculated with Eq.8 and Eg.9. The parameters for the calculation are those in Figure 3 in the main manuscript, obtained with Eq. 11 and Eq. 12.

6. Additional representation of R and C as a function of temperature.

Figure S10 shows C_{gb} variations with temperature. No significant change in C_{gb} can be observed, in part due to the low signal-to-noise ratio in the high-frequency region of the Nyquist plot. C_{gb} is calculated from the approximate size of the high-frequency tail with Eqs. 8 and 9.

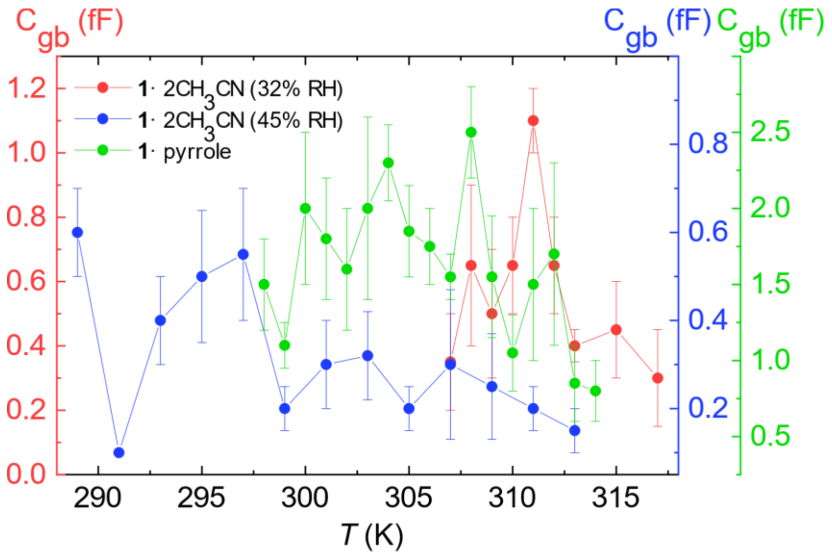

Figure S 10 C_{gb} variations with temperature for **1**·2CH3CN at 32% RH (red dots, sample 3), at 45% RH (blue dots, sample 4), and the pyrrole-substituted **1**·pyrrole crystal (green dots, sample 6).

Figure S11 shows a) R_c, b) C_c, and c) R_{gb} d) C_{gb} as a function of temperature (as in Figure 3 of the main manuscript) and normalized by the critical temperature for the **1**·2CH_3CN at 32 % RH (red dots), the **1**·2CH_3CN crystal at 45 % RH (blue dots) and pyrrole-substituted **1**·pyrrole crystal (green dots).

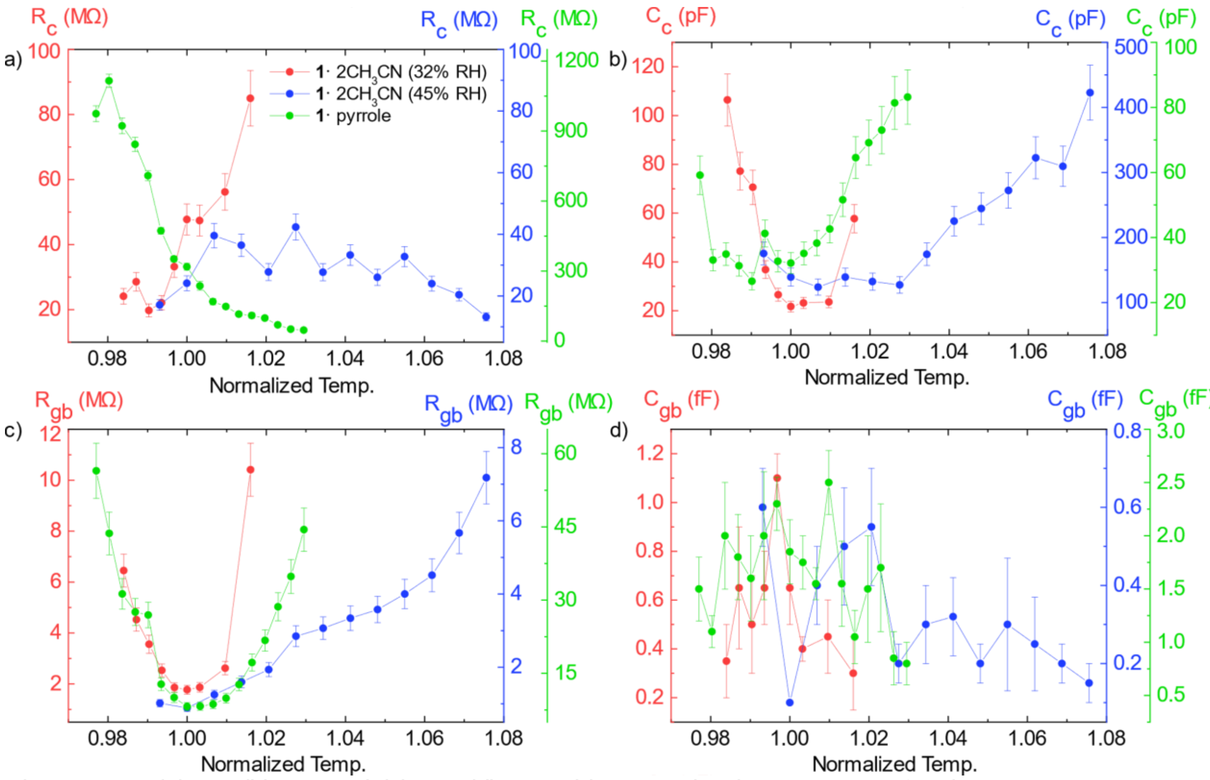

Figure S 11 (a) R_c, (b) C_c, and (c) R_{gb} (d) C_{gb} with normalized temperature for the **1**·2CH_3CN at 32 % RH (red dots sample 3), **1**·2CH_3CN at 45 % RH (blue dots, sample 4) and pyrrole-substituted **1**·pyrrole (green dots, sample 6), respectively.

7. Frequency spectrum of G and B of pyrrole-substituted crystal

Frequency dependent AC admittance spectroscopy on a pyrrole-substituted **1**·pyrrole crystal has been carried out at different temperatures. Figure S12 shows G and B of sample 6 measured as a function of frequency (5 Hz < $\omega/2\pi$ < 510 kHz). At a fixed temperature, G increases with ω and begins to saturate at high frequencies (~10^4 Hz) however B develops a peak at around 10^3 Hz, additionally an increment in B appears at high frequencies (> 10^5 Hz).

Figure S13 shows G and B measured in three different **1**·pyrrole samples. The main features are qualitatively reproduced.

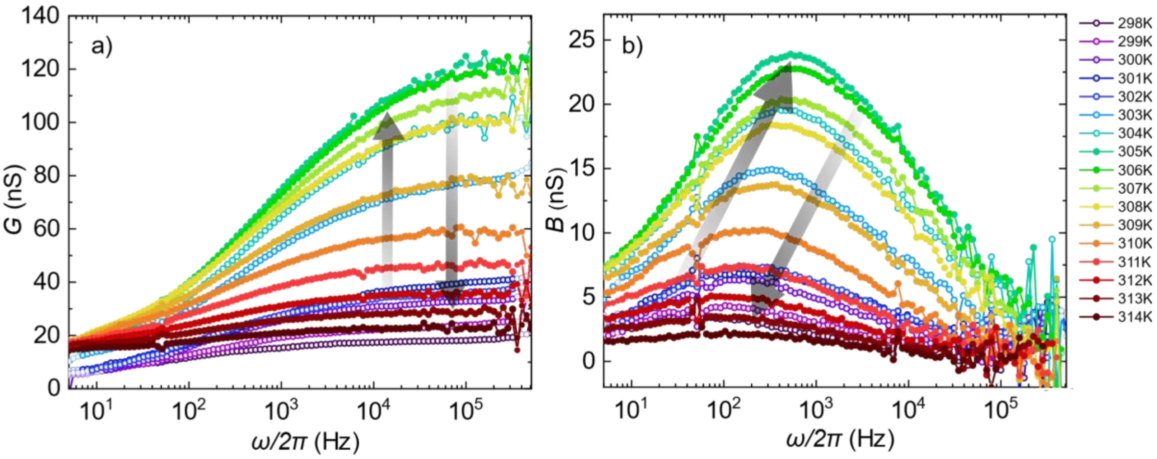

Figure S 12 a) Conductance *G* and b) susceptance *B* measured as a function of the temperature *T* of pyrrole-substituted crystal.

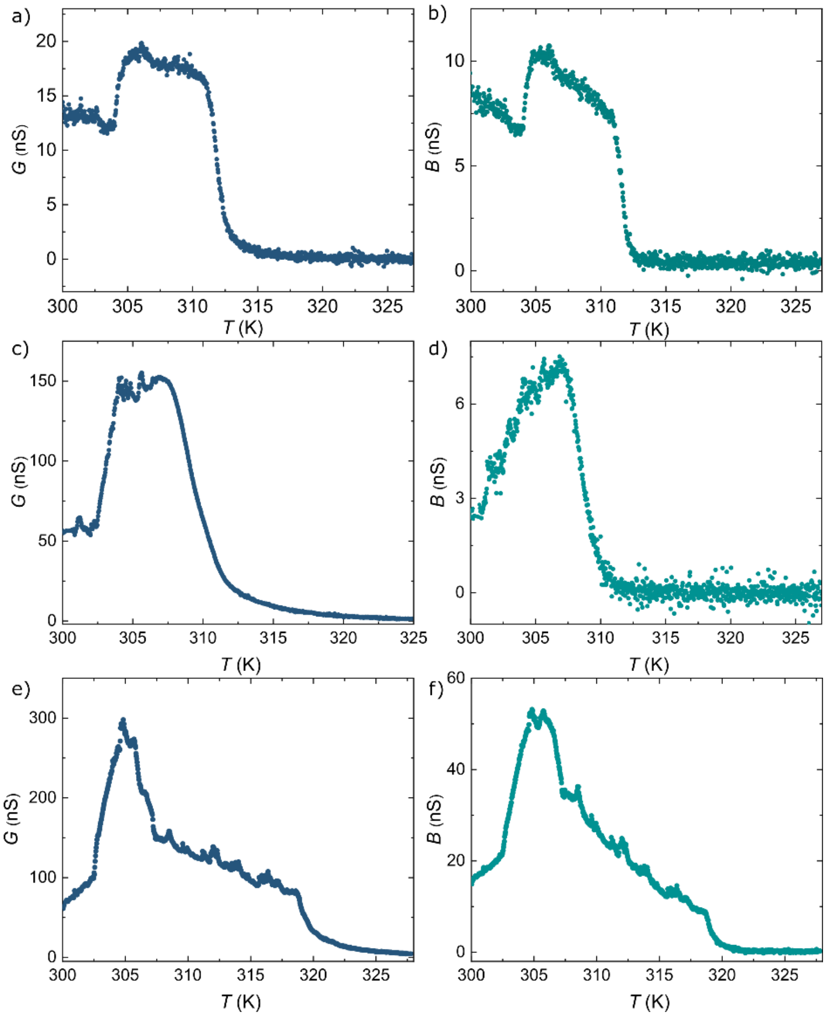

Figure S 13. *G* and *B* measured as a function of the temperature in three different **1**·pyrrole samples.

8. Time constant , equivalent impedance Nyquist plots and comparative of the conductance of the materials

Figure S14 shows the time constant ($\tau = R_e C_c$) calculated for the **1**·2CH_3CN at 32 % RH (red dots, sample 3), the **1**·2CH_3CN at 45 % RH (blue dots, sample 4), and pyrrole-substituted (green dots, sample 6) with temperature from their respective Nyquist plot. In all cases, τ decreases until critical temperature then starts to increase.

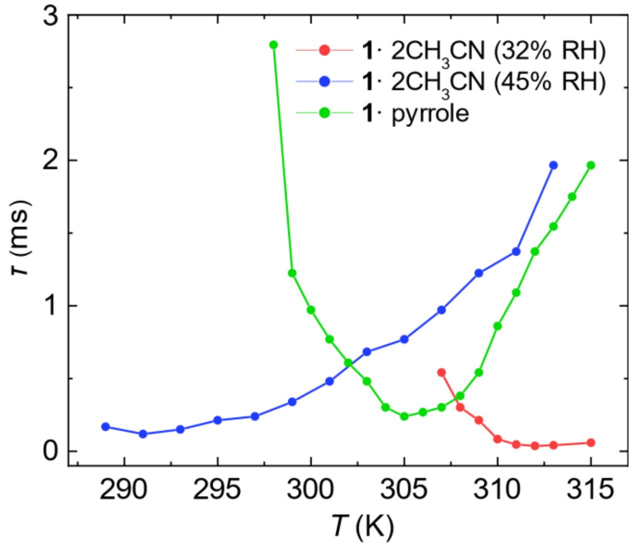

Figure S 14 Time constant τ for a) 32% RH (red dots, sample 3), b) 45% RH (blue dots, sample 4), c) pyrrole-substituted, **1**·pyrrole (green dots, sample6) with temperature.

Figure S 15 shows the equivalent Nyquist plots of the impedance calculated from the experimental admittance in a) Figure 2c (1·2CH3CN, 32%RH) b) Figure 4a (**1**·2CH3CN, 45%RH) and c) Figure 4d (**1**·pyrrole) in the main manuscript.

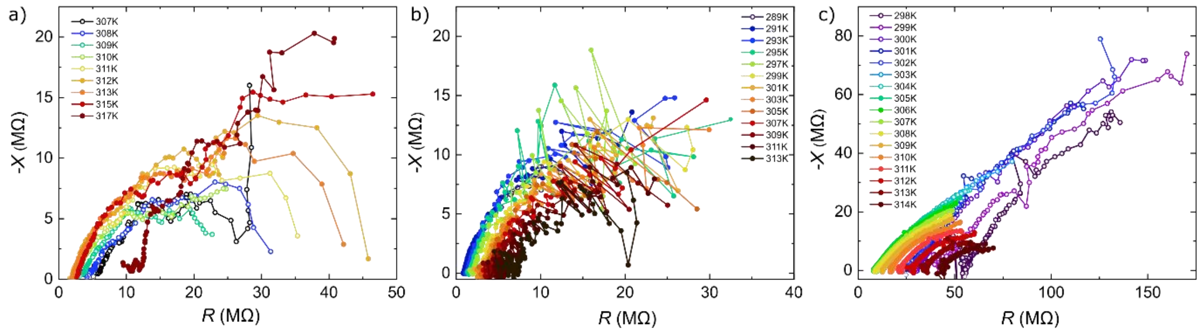

Figure S 15 Equivalent Nyquist plots of the impedance calculated from the admittance in a) Figure 2c (1·2CH_3CN, 32%RH) b) Figure 4a (1·2CH_3CN, 45%RH) and c) Figure 4d (1·pyrrole) in the main manuscript

The conductance at the peak obtained for the three different cases discussed in the manuscript is: 1·2CH_3CN (32% RH): 42.3 nS (11.6 µS/cm), 1·2CH_3CN (45% RH): 83.8 nS (9.42 µS/cm), 1·pyrrole: 17.6 nS (0.70 µS/cm). Note that these values may be heavily dependent on the aspect-ratio characteristics of each crystal

9. Synthesis of $_\infty\{[Fe(H_2O)_2(CH_3CN)_2(pyrazine)](BF_4)_2\cdot(CH_3CN)_2\}$ (1·2CH$_3$CN) and $_\infty\{[Fe(H_2O)_2(CH_3CN)_2(pyrazine)](BF_4)_2\cdot pyrrole\}$ (1·pyrrole)

As previously reported[1], **1**·2CH_3CN was synthetized at room temperature, dissolving 0.64 mmol of $Fe(BF_4)_2\cdot 6H_2O$ in 2.5 ml of acetonitrile and adding it drop by drop to a solution of 1.2 mmol of pyrazine in 2.5 mL of acetonitrile. The resulting solution was stirred for 15 minutes and filtered. After one day, yellow crystals appeared.

Anal. calcd for **1**·2$CH_3CN\cdot 0.6H_2O$ (**1**·2CH_3CN): C 27.69, H 4.10, N 16.14; found C 27.85, H 3.72, N 15.77.

FTIR **1**·2CH_3CN (cm^{-1}): v = 3481 (w; v(OH)), 2309 (w; v(C≡N)), 2281 (w; v(C≡N)), 1642 (m; v(CC)), 1488 (w), 1423 (s; δ(CH)), 1366 (w), 1289 (w), 1058 (s), 1002 (s, v(BF4)), 817 (s; δ(ring)), 766 (m; δ(CCN)), 589 (w), 518 (w), 466 (m).

Sample **1**·pyrrole was synthesized according to the following procedure. A screw vial for chromatography (diameter 12 mm, height 32 mm) displaying 10 mg of **1**·2CH_3CN was placed in a clear glass vial (diameter 27 mm, height 55 mm) containing 0.1 ml of pyrrole. The glass vial was sealed and kept at room temperature overnight to allow the gas-solid reaction between the VOC and **1**.

Anal calcd for **1**·$C_4H_5N\cdot 0.4CH_3CN\cdot 0.35H_2O$ (**1**·pyrrole): C 29.71, H 4.07, N 14.62; found C 29.49, H 4.01, N 14.47.

FTIR **1**·pyrrole (cm^{-1}): v = 3404 (m; v(OH); $v(NH)_{pyrrole}$), 3128 (w; v(CH)), 2950 (w), 2304 (w; v(C≡N)), 2276 (w; v(C≡N)), 1640 (w; v(CC)), 1530 (w), 1422 (m; δ(CH)), 1371 (w), 1287 (w), 1045 (w, $v(ring)_{pyrrole}$), 1011 (b; v(BF4)), 1010 (w, $v(ring)_{pyrrole}$), 883 (w), 814 (m; $\delta(ring)_{oop,pyrazine}$), 731 (s; $\delta(ring)_{oop,pyrrole}$), 553 (w), 520 (w), 492 (w), 456 (m).

The elemental analysis for **1**·pyrrole fit properly with the TGA analysis (see below), where the two weight losses are consistent with the loss of 0.4CH_3CN, 1 pyrrole and 0.35 H_2O molecules per unit formula.

10. Thermogravimetric analyses

As expected, **1**·2CH_3CN and **1**·pyrrole display different thermal properties, as can be observed in the thermogravimetric analyses (Figure S16-S18). In **1**·2CH_3CN, a first weight loss (15.73 %) can be observed between 315 K and 345 K, that corresponds to the loss of two acetonitrile molecules (16.08 %). This polymer decomposes completely above 440 K.

However, in **1**·pyrrole, two weight losses can be seen. The first one, (3.80 %), between 340 K and 356 K corresponds to the loss of 0.4 CH_3CN molecules (3.6 %) and fits well with the evaporation temperature of acetonitrile, and the second one (14.09 %) can be seen between 373 K and 440 K and fits well with the evaporation temperature of pyrrole, it can be therefore assigned to the loss of a pyrrole and 0.35 H_2O molecules (14.09 %). Note that the peaks appearing in the conductance happen at lower temperatures where the TGA is not sensitive enough to observe changes.

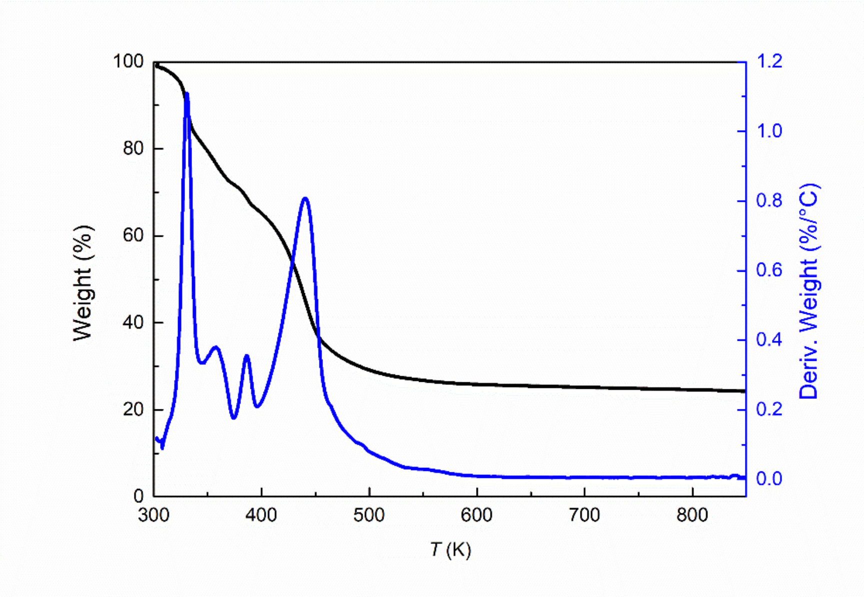

Figure S 16 Thermogravimetric analysis of **1**·2CH_3CN between 303 K and 873 K.

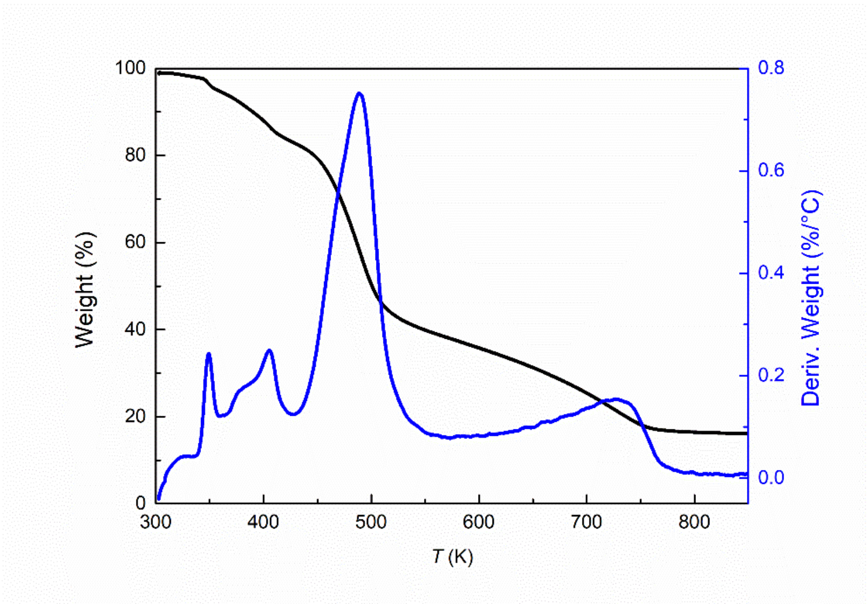

Figure S 17 Thermogravimetric analysis of **1**·pyrrole between 303 K and 873 K.

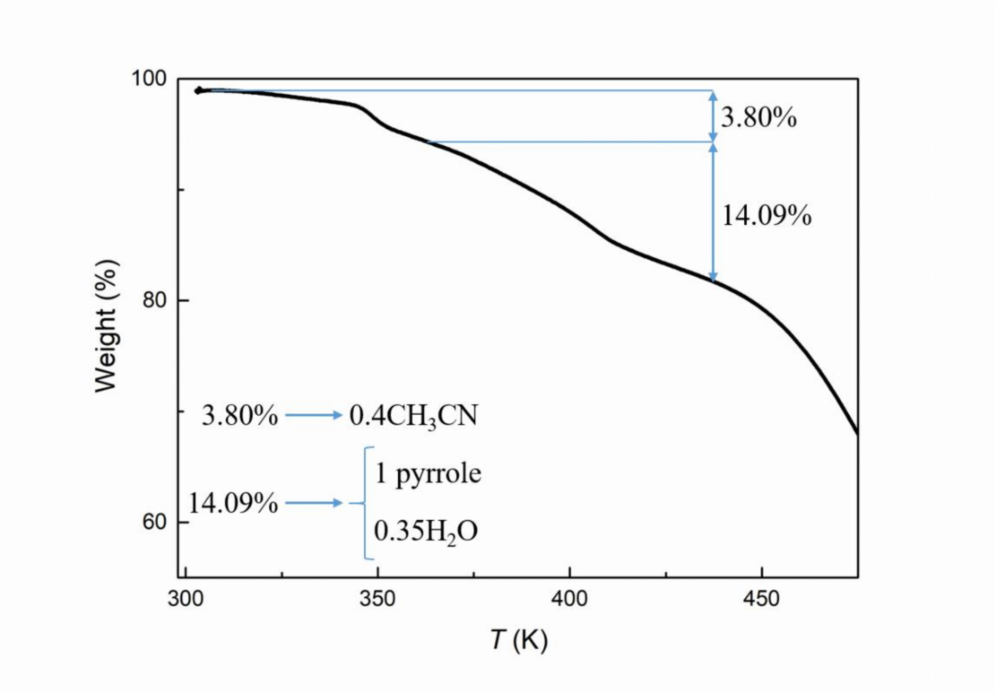

Figure S 18 Thermogravimetric analysis of **1**·pyrrole between 303 K and 475 K

11. Optical Reflectivity upon pyrrole exposure

Figure S19 shows the color variation of a **1**·2CH_3CN crystal upon exposure to pyrrole at RT, and the crystal color changes from yellow to dark green.

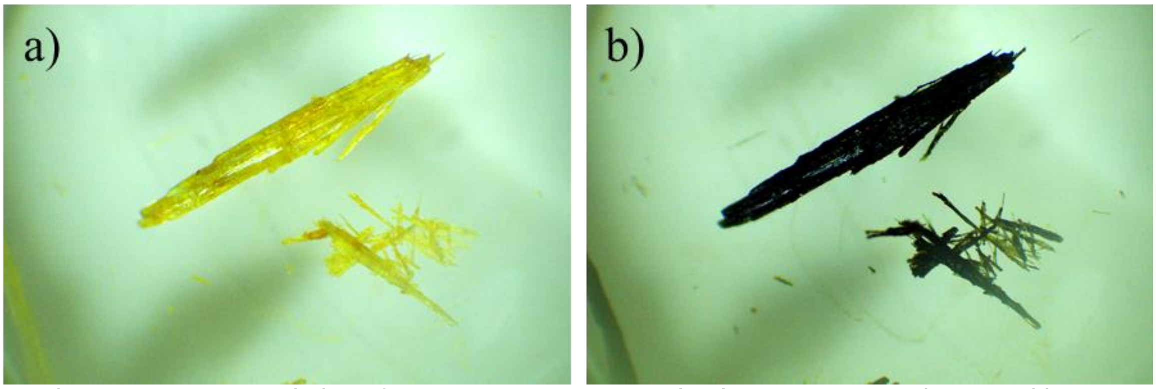

Figure S 19 Color variation of a **1**·2CH_3CN crystal turning into **1**·pyrrole under exposition to pyrrole. Images taken at time zero (a) and 14 hours (b).

The optical reflectivity of the crystal was measured as the exposure time progresses, see Figure S20. The OR value starts decreasing almost immediately, reaching a small plate after 1 hour. The value continues decreasing progressively until it reaches a minimum after a total of 4 hours. This value remains constant if exposure continues (up to 14 hours).

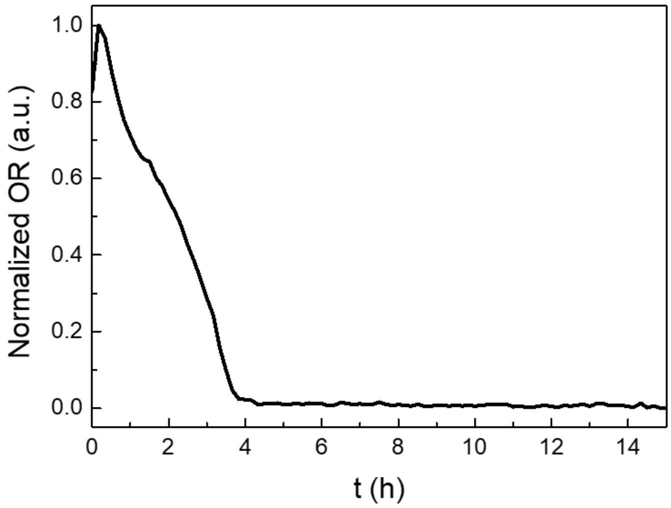

Figure S 20. Optical reflectivity of **1**·2CH_3CN crystal turning into **1**·pyrrole under exposition to pyrrole.

.

12. Fourier Transform Infrared Spectroscopy (FTIR)

Figure S21 shows the infrared absorption spectra of **1**·2CH_3CN (yellow), pyrrole (brown) and **1**·pyrrole. For this last one, the characteristic bands of the backbone structure of **1** are represented in yellow, while the vibration bands that can be assigned to the inclusion of pyrrole are colored in brown.

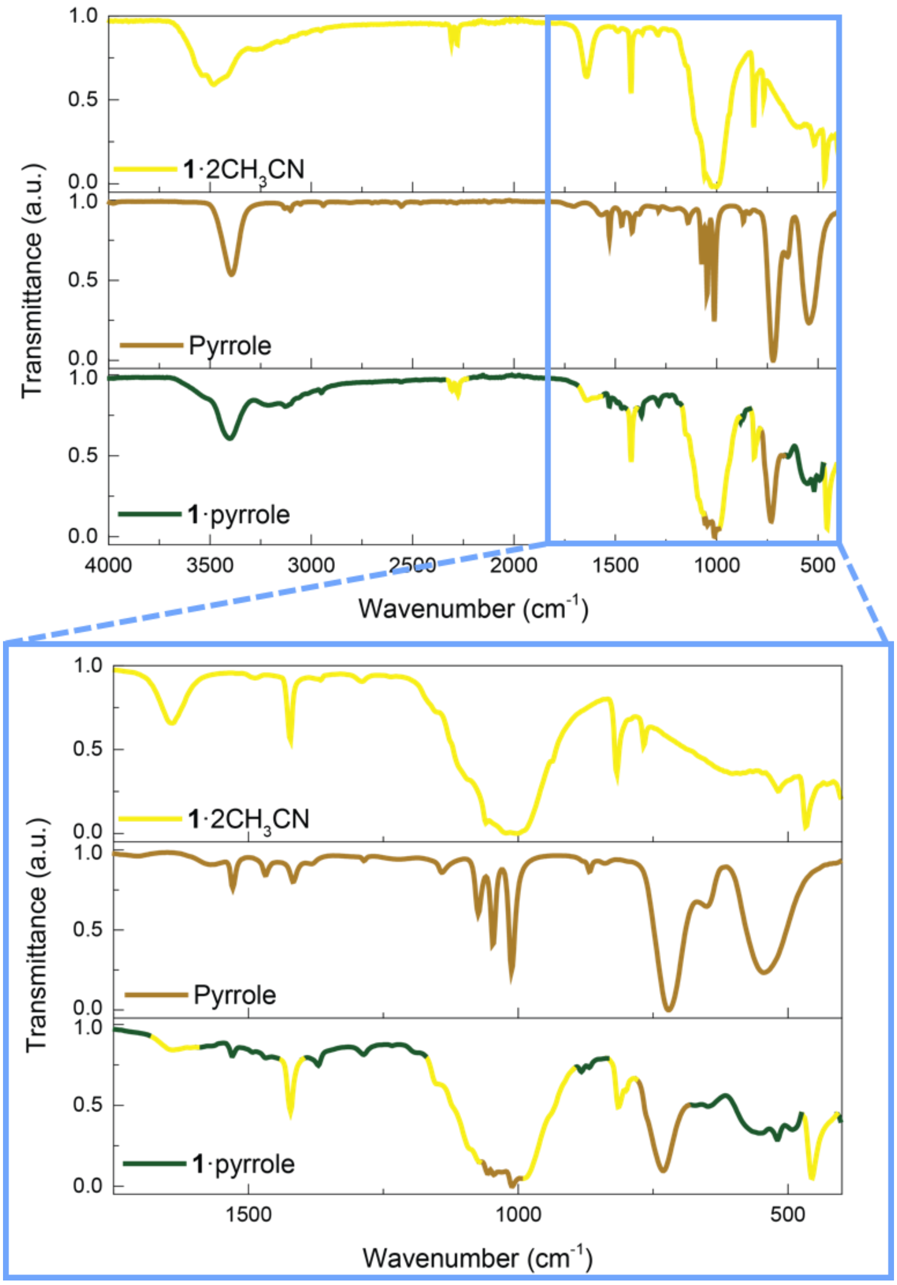

Figure S 21. IR spectrum of 1·2CH_3CN, pyrrole and **1**·pyrrole between 4000 cm^{-1} and 400 cm^{-1} A zoom for the 1750-400 cm^{-1} range can be observed at the bottom.

13. Powder X-ray Diffraction (PXRD) study

All attempts to resolve the crystal structure of **1**·pyrrole by single crystal X-Ray diffraction have failed, so PXRD analyses were performed on this compound as well as on the starting material, **1**·2CH_3CN. The key observation of this study is that both diffraction patterns are different, thereby providing further confirmation of the occurrence of structural modifications (See Figure S22).

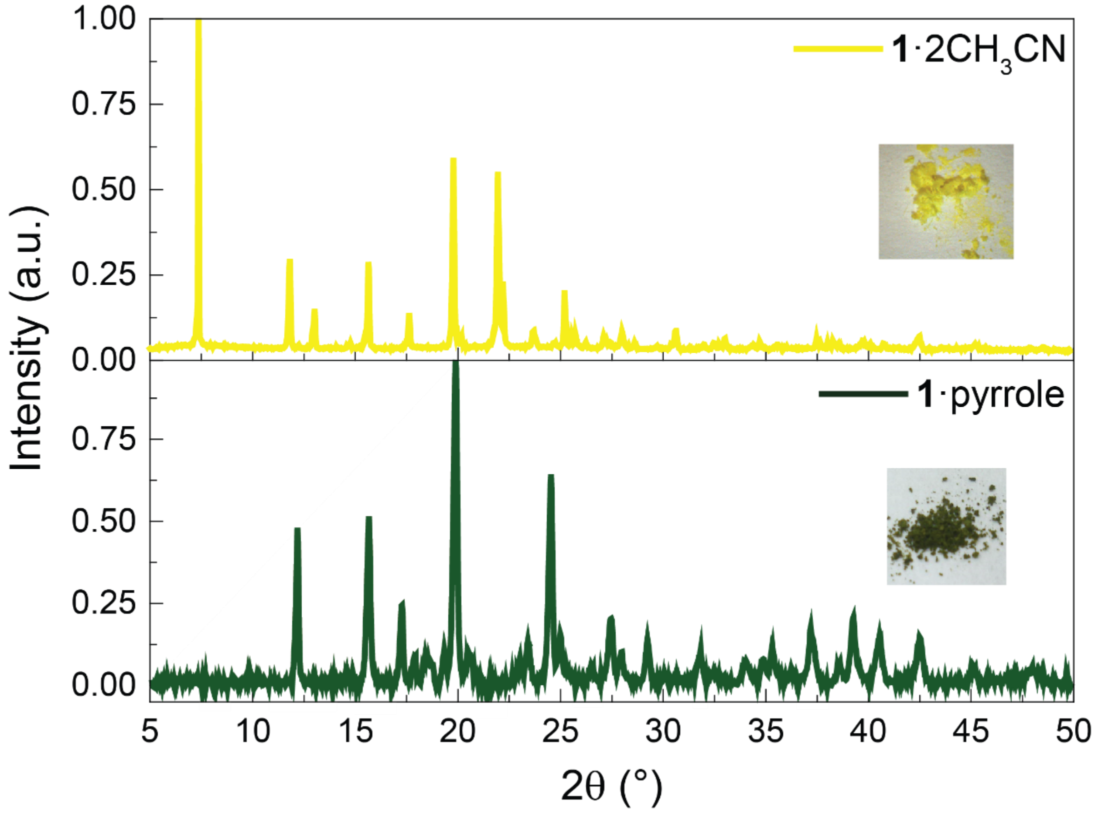

Figure S 22. PXRD pattern of **1**·2CH_3CN and **1**·pyrrole. Inset shows a picture of the powders.

14. Density functional theory calculations

Density Functional Theory (DFT) calculations are performed using QUANTUM ESPRESSO[15]. Exchange and correlation is treated using the PBE (Perdew-Burke-Ernzerhof) functional[16]. For a better description of the electronic density[17] we employ PBE+U with a Hubbard *U* correction of 5 eV applied to d electrons. The PBE+D2[18] approach is used to account for weak interactions. Ultrasoft Rappe-Rabe-Kaxiras-Joannopoulos pseudopotentials[19,20] from the pslibrary are used, and we set energy cutoffs of 50 Ry and 500 Ry for the representation of the wavefunction and charge density, respectively. Geometrical optimizations are performed for **1** and **1**·2CH_3CN and **1**·2H_2O compounds (*i.e.* empty, with interstitial acetonitrile and water, respectively) until forces are less than 0.004 eV Å^{-1} and the stress is less than 0.015 kbar.

A unit cell containing 204, 156, and 180 atoms is used respectively for **1**, **1**·2CH_3CN, and **1**·2H_2O. The Fe atoms are in high spin configuration in accordance with experiment and the use of the unit cell implies a ferromagnetic alignment of the Fe ions along the chains (*x* direction). A Monkhorst-Pack grid of 3x1x1 is used for geometrical optimization. Upon acetonitrile and water uptake, the compound exhibits a volume expansion by 26% and 14%, respectively. The lattice parameters of **1**, **1**·2CH_3CN, and **1**·2H_2O are reported in the Table S2.

Table S2. Lattice parameters of the optimized geometries computed using PBE+U+D2.

**compound**	***a* (Å)**	***b* (Å)**	***c* (Å)**	**α (º)**	**β(º)**	**γ (º)**
**1**	7.218	10.512	22.719	91.411	90.091	88.050
**1**·2CH_3CN	7.215	12.505	24.068	89.636	90.016	90.013
**1**·2H_2O	7.142	12.041	22.842	90.081	89.896	92.122

For the **1**·2CH_3CN complex, we optimize the geometry starting from the experimental coordinates. For **1**·2H_2O, we place the guest water molecules forming an hydrogen bond with the water that is coordinated to Fe. We have tried several orientations and we report only the result for the the lowest energy configuration. This corresponds to adsorbate molecules forming a OH···O bond of 1.64 Å with the coordinated water molecule and pointing the OH groups towards the BF_4 ions as illustrated in the Figure S23. For each molecule the two OH···F bond distances are ~1.94 Å and 1.88 Å.

We also attempted a full geometrical optimization of **1**·pyrrole but were unable to converge the calculations possibly owing to the large steric hindrance within the material.

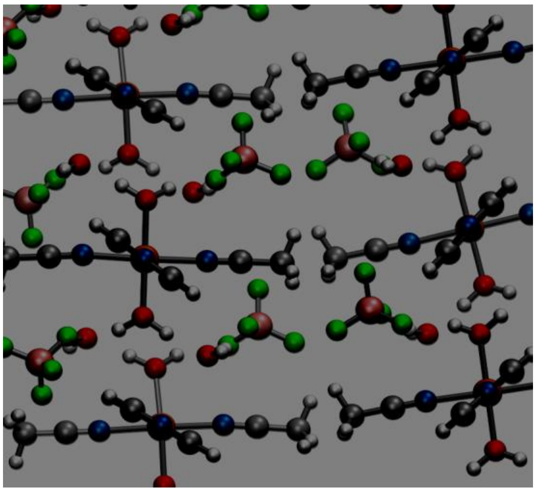

Figure S 23. Illustration of the geometrically optimized **1**·2H_2O crystal. The guest water molecules form an OH bond with the coordinated water and points towards the BF4 ions possibly via electrostatic/hydrogen bond. Color code: Fe: orange; N: blue; O: red; B: pink; F: green; C: silver; H: white

15. Bibliography

[1] E. Resines-Urien, E. Burzurí, E. Fernandez-Bartolome, M. Á. García García-Tuñón, P. De La Presa, R. Poloni, S. J. Teat, J. S. Costa, *Chem. Sci.* **2019**, *10*, 6612.

[2] B. Xiao, P. J. Byrne, P. S. Wheatley, D. S. Wragg, X. Zhao, A. J. Fletcher, K. M. Thomas, L. Peters, J. S. O. Evans, J. E. Warren, W. Zhou, R. E. Morris,

Nat. Chem. **2009**, *1*, 289.
[3] A. Lennartson, P. Southon, N. F. Sciortino, C. J. Kepert, C. Frandsen, S. Mørup, S. Piligkos, C. J. McKenzie, *Chem. - A Eur. J.* **2015**, *21*, 16066.
[4] E. Coronado, M. Giménez-Marqués, G. M. Espallargas, L. Brammer, *Nat. Commun.* **2012**, *3*, DOI 10.1038/ncomms1827.
[5] J. Miguel-donet, J. López-cabrelles, C. Galve, E. Coronado, *Chem. Eur. J.* **2018**.
[6] S. Rodrïguez-Jimenez, H. L. C. Feltham, S. Brooker, *Angew. Chemie - Int. Ed.* **2016**, *55*, 15067.
[7] C. Knoll, D. Müller, M. Seifried, G. Giester, J. M. Welch, W. Artner, K. Hradil, M. Reissner, P. Weinberger, *Dalt. Trans.* **2018**, *47*, 5553.
[8] A. Galet, M. C. Muñoz, J. A. Real, *Chem. Commun.* **2006**, *41*, 4321.
[9] A. Tarassoli, V. Nobakht, E. Baladi, L. Carlucci, D. M. Proserpio, *CrystEngComm* **2017**, *19*, 6116.
[10] A. L. Spek, *Acta Crystallogr. Sect. E Crystallogr. Commun.* **2020**, *E76*, 1.
[11] O. V. Dolomanov, L. J. Bourhis, R. J. Gildea, J. A. K. Howard, H. Puschmann, *J. Appl. Crystallogr.* **2009**, *42*, 339.
[12] J. Sánchez Costa, S. Rodríguez-Jiménez, G. A. Craig, B. Barth, C. M. Beavers, S. J. Teat, G. Aromí, *J. Am. Chem. Soc.* **2014**, *136*, 3869.
[13] G. Aromí, C. M. Beavers, J. S. Costa, G. A. Craig, G. M. Espallargas, A. Orera, O. Roubeau, *Chem. Sci.* **2016**, *7*, 2907.
[14] P. R. Bueno, J. A. Varela, E. Longo, *J. Eur. Ceram. Soc.* **2007**, *27*, 4313.
[15] P. Giannozzi, S. Baroni, N. Bonini, M. Calandra, R. Car, C. Cavazzoni, D. Ceresoli, G. L. Chiarotti, M. Cococcioni, I. Dabo, A. Dal Corso, S. De Gironcoli, S. Fabris, G. Fratesi, R. Gebauer, U. Gerstmann, C. Gougoussis, A. Kokalj, M. Lazzeri, L. Martin-Samos, N. Marzari, F. Mauri, R. Mazzarello, S. Paolini, A. Pasquarello, L. Paulatto, C. Sbraccia, S. Scandolo, G. Sclauzero, A. P. Seitsonen, A. Smogunov, P. Umari, R. M. Wentzcovitch, *J. Phys. Condens. Matter* **2009**, *21*, 395502.
[16] J. P. Perdew, K. Burke, M. Ernzerhof, *Phys. Rev. Lett.* **1996**, *77*, 3865.
[17] L. A. Mariano, B. Vlaisavljevich, R. Poloni, *J. Chem. Theory Comput.* **2021**, *17*, 2807.
[18] S. Grimme, *J. Comput. Chem.* **2006**, *27*, 1787.
[19] A. M. Rappe, K. M. Rabe, E. Kaxiras, J. D. Joannopoulos, *Phys. Rev. B* **1990**, *41*, 1227.
[20] A. M. Rappe, K. M. Rabe, E. Kaxiras, J. D. Joannopoulos, *Phys. Rev. B* **1991**, *44*, 13175.
